# Endogenous Plasmids and Chromosomal Genome Reduction in the *Cardinium* Endosymbiont of *Dermatophagoides farinae*

**DOI:** 10.1128/msphere.00074-23

**Published:** 2023-03-20

**Authors:** Qing Xiong, Cathy Sin-Hang Fung, Xiaojun Xiao, Angel Tsz-Yau Wan, Mingqiang Wang, Pavel Klimov, Yaning Ren, Kevin Yi Yang, Jan Hubert, Yubao Cui, Xiaoyu Liu, Stephen Kwok-Wing Tsui

**Affiliations:** a School of Biomedical Sciences, The Chinese University of Hong Kong, Hong Kong; b Hong Kong Bioinformatics Centre, The Chinese University of Hong Kong, Hong Kong; c Shenzhen Key Laboratory of Allergy and Immunology, School of Medicine, Shenzhen University, China; d Department of Biological Sciences, Purdue University, West Lafayette, Indiana, USA; e Clinical Research Center, The Affiliated Wuxi People’s Hospital of Nanjing Medical University, Wuxi, China; f Faculty of Agrobiology, Food and Natural Resources, Czech University of Life Sciences Prague, Prague, Czechia; g Centre for Microbial Genomics and Proteomics, The Chinese University of Hong Kong, Hong Kong; University of Wisconsin-Madison

**Keywords:** *Cardinium* endosymbiont, chromosomal genome reduction, endogenous plasmid, host-associated bacteria

## Abstract

*Cardinium* bacteria are well known as endosymbionts that infect a wide range of arthropods and can manipulate host reproduction to promote their vertical transmission. As intracellular bacteria, *Cardinium* species undergo dramatic genome evolution, especially their chromosomal genome reduction. Although *Cardinium* plasmids have been reported to harbor important genes, the role of these plasmids in the genome evolution is yet to be fully understood. In this study, 2 genomes of *Cardinium* endosymbiont bacteria in astigmatic mites were *de novo* assembled, including the complete circular chromosomal genome of *Cardinium* sp. DF that was constructed in high quality using high-coverage long-read sequencing data. Intriguingly, 2 circular plasmids were assembled in *Cardinium* sp. DF and were identified to be endogenous for over 10 homologous genes shared with the chromosomal genome. Comparative genomics analysis illustrated an outline of the genome evolution of *Cardinium* bacteria, and the in-depth analysis of *Cardinium* sp. DF shed light on the multiple roles of endogenous plasmids in the molecular process of the chromosomal genome reduction. The endogenous plasmids of *Cardinium* sp. DF not only harbor massive homologous sequences that enable homologous recombination with the chromosome, but also can provide necessary functional proteins when the coding genes decayed in the chromosomal genome.

**IMPORTANCE** As bacterial endosymbionts, *Cardinium* typically undergoes genome reduction, but the molecular process is still unclear, such as how plasmids get involved in chromosome reduction. Here, we *de novo* assembled 2 genomes of *Cardinium* in astigmatic mites, especially the chromosome of *Cardinium* sp. DF was assembled in a complete circular DNA using high-coverage long-read sequencing data. In the genome assembly of *Cardinium* sp. DF, 2 circular endogenous plasmids were identified to share at least 10 homologous genes with the chromosomal genome. In the comparative analysis, we identified a range of genes decayed in the chromosomal genome of *Cardinium* sp. DF but preserved in the 2 plasmids. Taken together with in-depth analyses, our results unveil that the endogenous plasmids harbor homologous sequences of chromosomal genome and can provide a structural basis of homologous recombination. Overall, this study reveals that endogenous plasmids participate in the ongoing chromosomal genome reduction of *Cardinium* sp. DF.

## OBSERVATION

**FIG 1:**
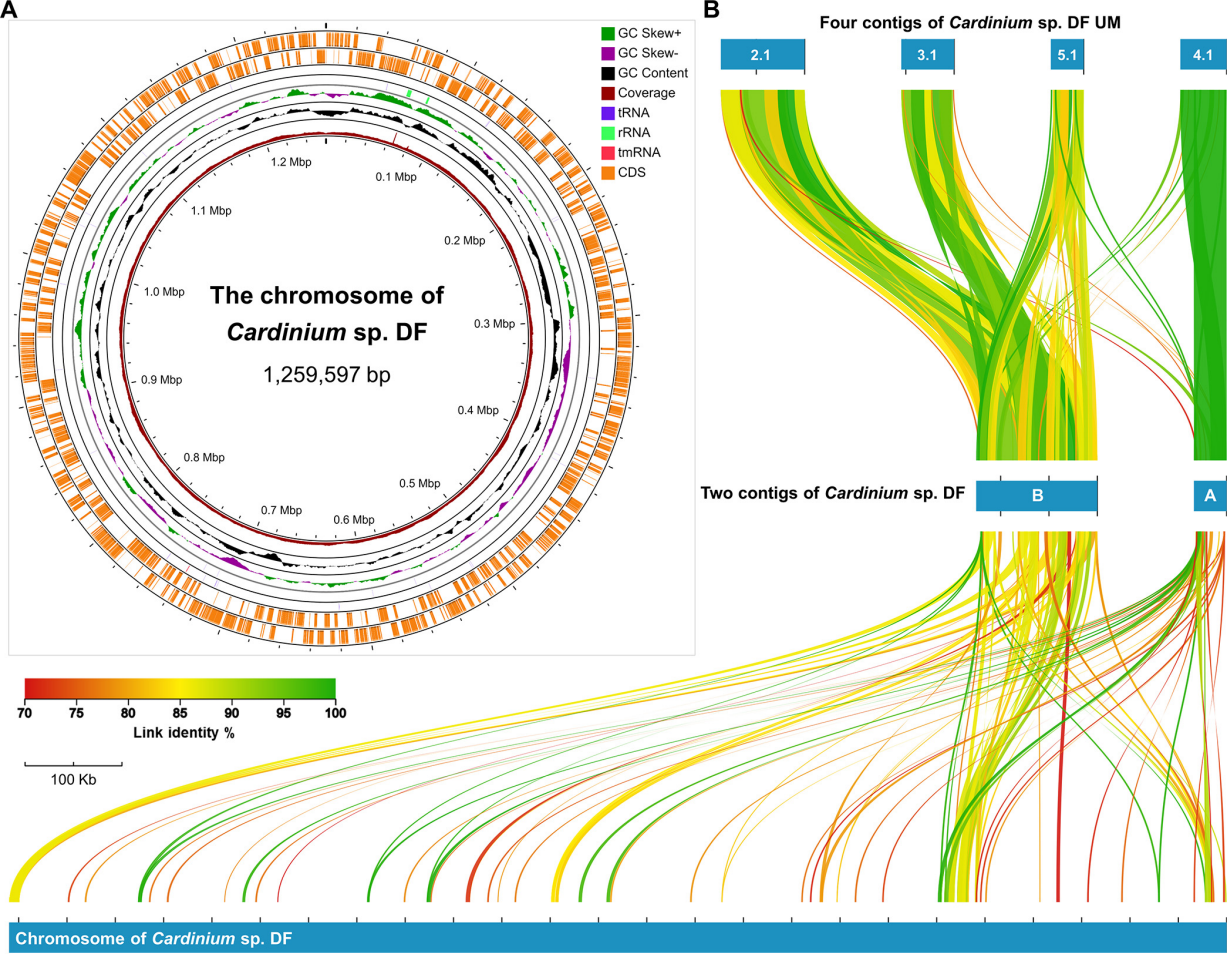
The chromosomal genome map and whole alignment of *Cardinium* sp. DF. (A) Circular chromosomal genome map of *Cardinium* sp. DF. The genome annotation was performed by Prokka and visualized by the online tool Proksee. The coverage was calculated with the Nanopore and PacBio long reads mapping, in which the height of the most internal layer was computed as (the site coverage)/(the highest coverage) and the full height indicated the highest coverage of 556 X. (B) Whole alignment of *Cardinium* sp. DF genome and other short contigs performed by AliTV and further filtered by 70% identity and 2-Kb link length. Two short contigs of *Cardinium* sp. DF and 4 short contigs of *Cardinium* sp. DF UM were aligned with the chromosomal genome of *Cardinium* sp. DF. Two contigs of *Cardinium* sp. DF are named contigs (subsequently confirmed as plasmids) (A and B). All the contigs of *Cardinium* sp. DF UM have a prefix of NZ_VMBH0100000 in the NCBI GenBank database.

*Cardinium* species belong to the family *Amoebophilaceae* of the CFB group bacteria, are a group of endosymbiont bacteria widely distributed among arthropods and can manipulate the reproductive system of host to promote their vertical transmission (1). With the advent of high-throughput sequencing technologies, genomic sequencing has become a primary tool for studying *Cardinium* endosymbionts. Using high-throughput sequencing data, we *de novo* assembled the genomes of *Cardinium* endosymbionts (Table S1) in 2 astigmatic mites, *Dermatophagoides farinae* and *Tyrophagus putrescentiae* (2, 3). The methods are described in Text S1. The *Cardinium* endosymbiont of *D. farinae*, *Cardinium* sp. DF, has a main chromosome of 1,259,597 bp in size, assembled in a single contig (Table S1 and Fig. 1A). The chromosome genome assembly and the annotated 1,198 proteins were assessed as 76.4% and 77.7% complete, respectively (Table S1).

10.1128/msphere.00074-23.1 TEXT S1 Supplemental methods. Download Text S1, DOCX file, 0.05 MB. Copyright © 2023 Xiong et al. 2023 Xiong et al. https://creativecommons.org/licenses/by/4.0/ This content is distributed under the terms of the Creative Commons Attribution 4.0 International license.

10.1128/msphere.00074-23.2 TABLE S1 Overview statistics of the genome assemblies of three *Cardinium* bacteria. Download Table S1, DOCX file, 0.01 MB. Copyright © 2023 Xiong et al. 2023 Xiong et al. https://creativecommons.org/licenses/by/4.0/ This content is distributed under the terms of the Creative Commons Attribution 4.0 International license.

It is difficult to assess assembly completeness or identify potential plasmids in endosymbiont genomes from metagenomic sequencing, because of the necessity to remove extraneous and presumable host contigs in all such data sets. This challenge can be further complicated by apparent horizontal gene transfer to hosts, leaving uncertainties about the cellular compartments of such bacterial genomes. A previous genome assembly of the *Cardinium* endosymbiont of *D. farinae* was constructed in 5 contigs, but only the longest contig was considered as the main chromosomal genome (4) (Table S1). To differentiate the 2 sequenced strains of the endosymbiont of *D. farinae*, the previous assembly (GenBank accession: GCF_007559345.1) was named *Cardinium* sp. DF UM because it was reported by the University of Michigan. The shared identity of the 16S rRNA sequences of DF and DF UM was 100%. The dot plot of 2 *Cardinium* sp. DF genome assemblies suggested that the genome was a circular DNA, in which our *de novo* assembly was a complete circular chromosome, while the assembly of *Cardinium* sp. DF UM was missing the 675,805 to 676,487 bp region in our assembly (Fig. S1A). Intriguingly, our genome assembly of *Cardinium* sp. DF was annotated with fewer protein-coding genes but higher completeness (Table S1). We confirmed that the low-quality assembly, especially in regions of repetitive bases, caused false-positive frameshifts and gene fission in the genome assembly of *Cardinium* sp. DF UM, which resulted in more genes but lower completeness. Although both genomes were assembled by third-generation sequencing (TGS) long reads (4), the lower sequencing coverage of *Cardinium* sp. DF UM resulted in its poorer assembly quality.

10.1128/msphere.00074-23.13 FIG S1 Dot plot of *Cardinium* sp. DF and phylogenetic analysis of *Cardinium* species. (A) The dot plot was generated by Gepard v2.1 with the chromosomal genome of *Cardinium* sp. DF and the longest contig of *Cardinium* sp. DF UM. The key junction sites were highlighted and labeled. The staggered feature between two chromosomal genomes suggested both were circular DNA and the region 675,805-676,487 of *Cardinium* sp. DF has no match in *Cardinium* sp. DF UM. (B) The phylogenetic tree was constructed based on 295 single-copy orthogroups. The annotated protein sequences of species were assigned into orthogroups by OrthoFinder v2.5.4. The identity matrix of *Cardinium* genomes were constructed by the online tool SIAS (Sequence Identity And Similarity) in default parameters and based on the protein sequence alignment of genes in the 295 single-copy orthogroups assigned by OrthoFinder. (C) The phylogenetic tree was constructed based on 16S rRNA sequences. All the source of those sequences were listed in Table S2. Both two phylogenetic trees were constructed by the program RAxML and edited by the online tool iTOL. (D) The whole-genome alignment of genome sequences was performed and presented by AliTV. The whole genome alignment of four *Cardinium* species was filtered by 70% identity and 5-Kb link length. The chromosomal genome of *Cardinium* sp. DF and only the longest contig NZ_VMBH01000001.1 of *Cardinium* sp. DF UM were used in this alignment. Download FIG S1, TIF file, 5.0 MB. Copyright © 2023 Xiong et al. 2023 Xiong et al. https://creativecommons.org/licenses/by/4.0/ This content is distributed under the terms of the Creative Commons Attribution 4.0 International license.

10.1128/msphere.00074-23.3 TABLE S2 NCBI GenBank accessions of the sequences used in this study. Download Table S2, DOCX file, 0.02 MB. Copyright © 2023 Xiong et al. 2023 Xiong et al. https://creativecommons.org/licenses/by/4.0/ This content is distributed under the terms of the Creative Commons Attribution 4.0 International license.

For the *Cardinium* endosymbiont of *T. putrescentiae*, *Cardinium* sp. TP, the genome was assembled into 914,750 bp and 33 contigs (Table S1) by next-generation sequencing (NGS) data. The completeness of the genome assembly and annotation was 73.6% and 76.4%, respectively. The 16S rRNA of *Cardinium* sp. TP was reported, but there is no available genome assembly (5). The assembly quality of *Cardinium* sp. DF was apparently much better than that of *Cardinium* sp. TP because of the higher sequencing coverage (108.6 X TGS only reads of *Cardinium* sp. DF, <8 X NGS reads of *Cardinium* sp. TP and even fewer TGS reads). Considering the unequal assembly qualities, we cannot conclude that *Cardinium* sp. DF possesses a larger genome size than *Cardinium* sp. TP.

Along with the single contig of *Cardinium* sp. DF chromosome (Fig. 1A), 2 short contigs were assembled and presumed to be extrachromosomal genetic elements (Fig. 1B). The 2 short contigs were subsequently confirmed to be endogenous plasmids. Similarly, 4 short contigs were assembled within the genome of *Cardinium* sp. DF UM (Fig. 1B). In the entire alignment (Fig. 1B), the short contig A was mapped to the contig, NZ_VMBH01000004.1 of *Cardinium* sp. DF UM in high quality, while the other contig B was variably mapped to the other 3 contigs of *Cardinium* sp. DF UM. Both contigs A and B were dispersedly mapped to the main chromosome, as shown in a wide range of highly conserved alignments (cutoff was set as 70% identity and 2-kb length) (Fig. 1B).

Phylogenetic analysis was performed for the 2 *de novo* assembled genomes of *Cardinium* endosymbionts, along with other published sequences (Table S2). To generate a high quality phylogenetic analysis, 10 genome assemblies of *Cardinium* and *Amoebophilus asiaticus* as outgroup were collected for their fewer than 50 assembled sequences (Table S2), annotated, and extracted with 295 single-copy orthogroups (OGs). However, *Cardinium* sp. TP was unexpectedly clustered with the *Cardinium* endosymbiont of *Sogatella furcifera*, *Cardinium* sp. *Sogatella furcifera* (Fig. S1B), in which their protein identity was as high as 95.62% (Fig. S1B). Among 9 *Cardinium* genomes, 2 from *Cardinium hertigii* were located as outgroups to 7 other *Cardinium* strains without official species names (Fig. S1B). The other phylogenetic tree based on 16S rRNA sequence was constructed with 4 additional sequences from oribatid mites, the sister group of astigmatic mites (Fig. S1C). Although the 2 *Cardinium* sp. DF were clustered with 3 from oribatid mites, the extremely long branches of the 2 from the oribatid mite *Microzetorchestes emeryi* impeded further discussion (Fig. S1C). Similar to the phylogenetic tree based on 295 single-copy OGs, *Cardinium* sp. TP clustered with *Cardinium* sp. *Sogatella furcifera*. The similarity among *Cardinium* genomes was further explored in whole genome alignment performed by AliTV (6) (Fig. S1D). The 2 available *Cardinium* sp. DF genomic sequences were mapped and aligned with high quality (Fig. S1D). The fragmented genome of *Cardinium* sp. TP could also be mapped to that of *Cardinium* sp. *Sogatella furcifera* with high quality (Fig. S1D). *Sogatella furcifera* is well known as an important pest species in rice, while the host of *Cardinium* sp. TP, *T. putrescentiae*, is a storage mite mainly infesting stored grains, including rice. The closely related living environments of their hosts suggested plant-mediated horizontal transmission of intracellular symbiont (7, 8), which could explain why *Cardinium* sp. TP shares a close phylogenetic relationship with *Cardinium* sp. *Sogatella furcifera* (8), but not *Cardinium* sp. DF from the closely related host, house dust mite *D. farinae*.

Comparative genomics provided an outline for the reductive genome evolution of *Cardinium* species. Two *Cardinium* sp. DF chromosomal genomes were assigned more OGs than others (Fig. S2A), which is consistent with their relatively larger genome sizes (Table S2). In the Venn diagram of 4 closely related *Cardinium* species (Fig. S2B), 581 OGs were conserved among the 4 genomes, and the genes of *Cardinium* sp. DF within these OGs were assigned to 529 functional Clusters of Orthologous Groups (COGs), in which the top 2 COG categories were “Translation, ribosomal structure, and biogenesis” (21.74%) and “Replication, recombination, and repair” (17.39%) (Fig. S2C). For the 288 OGs of 2 *Cardinium* sp. DF not found in the genomes of *Cardinium* sp. TP and *Cardinium* sp. *Sogatella furcifera*, the genes of *Cardinium* sp. DF were assigned to 73 functional COGs (Fig. S2D), and the COG “Replication, recombination and repair” ranked as the second abundant. Both the high abundances of “Replication, recombination and repair” were mainly contributed by a wide range of DNA rearrangement-related genes including transposases. Considering these DNA rearrangement-related genes are not pseudogenes or essential functional genes, we proposed that these *Cardinium* genomes were undergoing rapid genome changes related to DNA rearrangement.

10.1128/msphere.00074-23.14 FIG S2 Comparative genomics analysis of *Cardinium* species. (A) Matrix of species-to-species overlapped orthogroup number. The protein sequences annotated in the genomes of nine *Cardinium* species and *Amoebophilus asiaticus* as outgroup were comparatively analyzed by the program OrthoFinder and assigned into orthogroups (OGs) according to the sequence similarity. The phylogenetic tree was adapted from Fig. S1B. (B) Venn diagram of four *Cardinium* bacteria. The orthogroup lists of the four *Cardinium* genomes were used to generate the Venn diagram, following the analysis of OrthoFinder. (C) Functional clusters of orthologous groups (COGs) of the genes of *Cardinium* sp. DF in the 581 conserved OGs among the four *Cardinium* genomes. (D) Functional COGs of the genes of *Cardinium* sp. DF in the 288 conserved OGs specific in the two *Cardinium* sp. DF genomes. The COG category was annotated by eggnog-mapper v2.1.5 and only the functional COGs over >2% were labeled. Download FIG S2, TIF file, 2.5 MB. Copyright © 2023 Xiong et al. 2023 Xiong et al. https://creativecommons.org/licenses/by/4.0/ This content is distributed under the terms of the Creative Commons Attribution 4.0 International license.

To further explore the genome evolution, we focused on the high quality genome of *Cardinium* sp. DF, taking together the main chromosome and its 2 short contigs (Fig. 1B) for analysis. In the dot plot of the short contig A of *Cardinium* sp. DF (Fig. S3A), the repeated terminal sequences (1 to 2,237 bp and 31,550 to 33,817 bp at 2 ends) suggested that it was a close circular DNA containing a pair of inverted repeats (approximately 920 bp). Therefore, the truncated part (1 to 31,550 bp) of contig A was considered a circular DNA and tentatively named Plasmid A (Fig. S3B). It was annotated with 28 protein-coding genes by Prokka (9) (Fig. S3B), in which only 3 genes were assigned functional names, including 2 transposase genes and the cell division protein PomZ. More functional gene annotations were performed by eggNOG-mapper (10, 11) (Table S3.1). Except for those DNA rearrangement-related genes, including relaxase, transposase and resolvase, 3 NUDIX hydrolase domain-containing genes (GPDKAJLJ_00012-14) were annotated as tandemly arrayed. A SymE toxin gene (GPDKAJLJ_00010) in the type I toxin-antitoxin system was also annotated, and this gene is located in the chromosomal genome of Escherichia coli (NCBI Gene ID: 949088). To further explore Plasmid A, all encoded proteins were compared against those annotated in its chromosomal genome, and 21 of 28 proteins could be well matched with an E-value cut-off 1E-6 (Table S4.1), especially for the Tn3 family transposase TnEc1, GPDKAJLJ_00027 that has multiple highly similar copies in the chromosomal genome (Table S4.2).

10.1128/msphere.00074-23.4 TABLE S3 EggNOG-mapper output of protein sequences of plasmids. Download Table S3, DOCX file, 0.03 MB. Copyright © 2023 Xiong et al. 2023 Xiong et al. https://creativecommons.org/licenses/by/4.0/ This content is distributed under the terms of the Creative Commons Attribution 4.0 International license.

10.1128/msphere.00074-23.5 TABLE S3.1 EggNOG-mapper output of protein sequences of Plasmid A. Download Table S3, DOCX file, 0.03 MB. Copyright © 2023 Xiong et al. 2023 Xiong et al. https://creativecommons.org/licenses/by/4.0/ This content is distributed under the terms of the Creative Commons Attribution 4.0 International license.

10.1128/msphere.00074-23.6 TABLE S3.2 EggNOG-mapper output of protein sequences of Plasmid B. Download Table S3, DOCX file, 0.03 MB. Copyright © 2023 Xiong et al. 2023 Xiong et al. https://creativecommons.org/licenses/by/4.0/ This content is distributed under the terms of the Creative Commons Attribution 4.0 International license.

10.1128/msphere.00074-23.7 TABLE S4 BLASTP output of annotated protein sequences of plasmids. The annotated protein sequences of plasmids were mapped to those of the chromosomal genome of *Cardinium* sp. DF using BLASTP with options “-evalue 1e-6 -outfmt 6 -max_target_seqs 1” and then sorted by bit score. “-max_target_seqs 1” was not set for GPDKAJLJ_00027 in Table 4.2. Download Table S4, DOCX file, 0.05 MB. Copyright © 2023 Xiong et al. 2023 Xiong et al. https://creativecommons.org/licenses/by/4.0/ This content is distributed under the terms of the Creative Commons Attribution 4.0 International license.

10.1128/msphere.00074-23.9 TABLE S4.1 BLASTP output of annotated protein sequences of Plasmid A. Download Table S4, DOCX file, 0.05 MB. Copyright © 2023 Xiong et al. 2023 Xiong et al. https://creativecommons.org/licenses/by/4.0/ This content is distributed under the terms of the Creative Commons Attribution 4.0 International license.

10.1128/msphere.00074-23.10 TABLE S4.2 BLASTP output of GPDKAJLJ_00027. Download Table S4, DOCX file, 0.05 MB. Copyright © 2023 Xiong et al. 2023 Xiong et al. https://creativecommons.org/licenses/by/4.0/ This content is distributed under the terms of the Creative Commons Attribution 4.0 International license.

10.1128/msphere.00074-23.11 TABLE S4.3 BLASTP output of annotated protein sequences of Plasmid B. Download Table S4, DOCX file, 0.05 MB. Copyright © 2023 Xiong et al. 2023 Xiong et al. https://creativecommons.org/licenses/by/4.0/ This content is distributed under the terms of the Creative Commons Attribution 4.0 International license.

10.1128/msphere.00074-23.15 FIG S3 Dot plot and genome map of Plasmid A and B of *Cardinium* sp. DF. (A) Dot plot of the contig A suggests the region 1-31,550 bp is a circular plasmid. This plasmid was named as Plasmid A of *Cardinium* sp. DF (Fig. 1A). (B) Circular genome map of Plasmid A of *Cardinium* sp. DF. The gene annotation was performed by Prokka and visualized by the online tool Proksee. (C) Dot plot of Plasmid B of *Cardinium* sp. DF revealed a wide range of repeated sequences. (D) Linear genome map of Plasmid B of *Cardinium* sp. DF. The gene annotation was performed by Prokka and only those genes with functional names were visualized by the online tool Proksee. (E) TGS long reads mapping of the junction site including the 2.5 kb sequences on two ends. Over 80 long reads spanned the junction site and suggested this is circular plasmid DNA. Reads mapping was performed by Integrative Genomics Viewer (IGV). Download FIG S3, TIF file, 7.0 MB. Copyright © 2023 Xiong et al. 2023 Xiong et al. https://creativecommons.org/licenses/by/4.0/ This content is distributed under the terms of the Creative Commons Attribution 4.0 International license.

For the other contig B, the dot plot showed complicated features with a wide range of repeated sequences (Fig. S3C). In the annotation, 8 protein-coding genes were identified as encoding transposases by Prokka (9) (Fig. S3D), and more functional annotations were performed by eggNOG-mapper (10, 11) (Table S3.2). Contig B was also suggested to be a circular DNA and tentatively named Plasmid B when the 2 ends could be connected by TGS long reads (Fig. S3E). Concurrent with that in Plasmid A, partial proteins encoded by Plasmid B present high similarities to those in the chromosomal genome, such as DIOAJDMK_00073 of Plasmid B, which shares 90.9% identity and 87.8% coverage with GPMKIAHG_00283 of the genome (Table S4.3). Therefore, the 2 plasmids were suggested to be endogenous plasmids that share homologous genes, including those DNA rearrangement-related (Table S3) with the chromosomal genome.

The sequencing coverage of contigs A and B was estimated to be 146.4 X and 231.0 X, respectively, while that of the chromosomal genome was only 108.6 X (in TGS reads). The higher coverages of the 2 short contigs confirmed that they were replicable extrachromosomal elements or plasmids in *Cardinium* sp. DF. Two *Cardinium* Plasmids were identified in *Cardinium* sp. Bemisia tabaci cBtQ1 and sp. *Encarsia pergandiella* (GenBank accessions HG422566.1 and HE983996.1, respectively) (12–14). In BLASTP comparison, at least 2 genes of Plasmid A (Table S3.1), GPDKAJLJ_00007 (putative transposase) and GPDKAJLJ_00028 (P-loop ATPase), shared high similarity (over 60% identity and 400 bit score) with those of Plasmid B and other 2 *Cardinium* plasmids. Therefore, we proposed that the 2 plasmids of *Cardinium* sp. DF shared the common ancestor with other *Cardinium* plasmids (13). In addition, the replication mechanism of the 2 endogenous plasmids is still unknown. Up to 6 genes of Plasmid B but none of Plasmid A were annotated with DNA replication-related primase activity (Table S3), which indicated that the 2 plasmids employed distinct replication machineries. Due to the lack of bacterial culture, we cannot further identify and classify the mechanisms of their replication.

**FIG 2:**
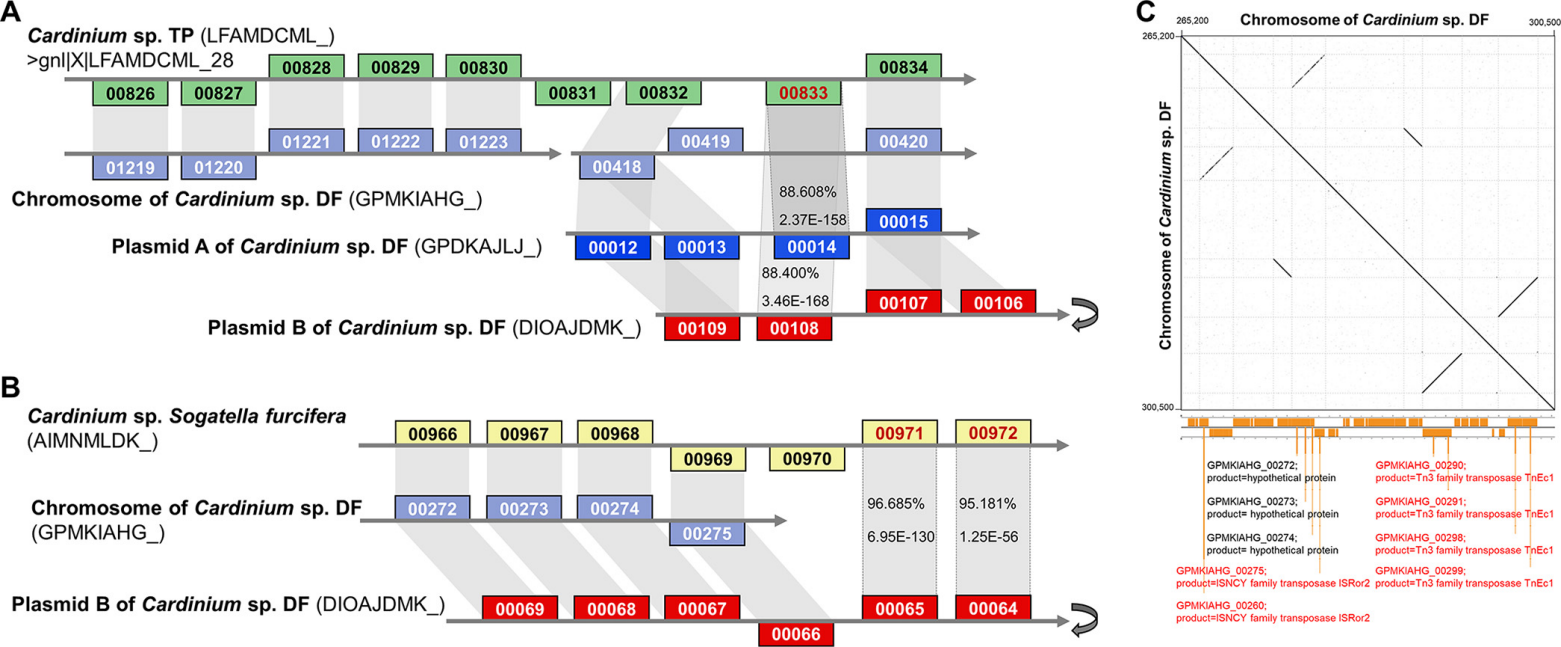
Alignment analysis of the chromosomal genome reduction in *Cardinium* sp. DF. (A) The NUDIX hydrolase gene LFAMDCML_00833 encoded by the contig gnl|X|LFAMDCML_28 of *Cardinium* sp. TP presented high percentages of identities, 88.608% and 88.400% with GPDKAJLJ_00014 of Plasmid A and DIOAJDMK_00108 of Plasmid B respectively; but has no homolog in the chromosomal genome of *Cardinium* sp. DF. The homologous gene of GPMKIAHG_ 00418 in the chromosome was split into 2 genes, GPDKAJLJ_00012 and GPDKAJLJ_00013 in Plasmid A. (B) The NUBPL iron-transfer P-loop NTPase gene AIMNMLDK_00971 and the hypothetical protein gene AIMNMLDK_00972 of *Cardinium* sp. *Sogatella furcifera* could not find homolog in the chromosomal genome of *Cardinium* sp. DF despite of the conserved synteny in their upstream genes; but shared high similarities with the 2 genes, DIOAJDMK_00065 and DIOAJDMK_00064 of Plasmid B, respectively. The genes above the gray backbone are located on the plus strand, while those below the backbone are on the minus strand. All homologous genes were connected by the gray bands. The gray turnover symbol means reverse complement. (C) Dot plot of *Cardinium* sp. DF chromosomal genome region (265,200 to 300,500 bp) containing 3 pairs of repeats. The partial gene annotations were adapted from Fig. 1A, and 3 inverted pairs of transposase genes were highlighted in red color. The dot plot was generated by Gepard v2.1.

The 2 extrachromosomal plasmids shared massive similar sequences with the chromosomal genome (Fig. 1B), and partially conserved sequences can be attributed to the expansion of mobile elements that are also important in homologous recombination, such as the Tn-3 family transposons that encode the multiple homologs of the Tn3 family transposase TnEc1, GPDKAJLJ_00027 (Table S3.2). In the further analysis, a range of unexpected gene conservation (Table S5) and gene synteny alignments (Fig. 2A and B) illuminated the molecular mechanism of the chromosomal genome reduction of *Cardinium* sp. DF. Genes, such as the N-terminal domain of reverse transcriptase DIOAJDMK_00062 of Plasmid B, decayed in the genome but remained in Plasmid B of *Cardinium* sp. DF (Table S5). We have excluded the artificial errors due to different sequencing and assembly methods and the candidate genes that could not find any conserved synteny in the chromosomal genome of *Cardinium* sp. DF. We did not take any pseudogenes into consideration.

10.1128/msphere.00074-23.12 TABLE S5 Candidate genes in chromosomal genome reduction of *Cardinium* sp. DF. These candidate genes encoded by Plasmid A or Plasmid B shared high percentage of identities with genes encoded by the genome of *Cardinium* sp. TP or *Cardinium* sp. *Sogatella furcifera* but have no homolog (E-value cutoff: 1E-6) from encoded genes of the chromosomal genome of *Cardinium* sp. DF. The gene description was annotated by eggnog-mapper v2.1.5. Download Table S5, DOCX file, 0.02 MB. Copyright © 2023 Xiong et al. 2023 Xiong et al. https://creativecommons.org/licenses/by/4.0/ This content is distributed under the terms of the Creative Commons Attribution 4.0 International license.

We selected 2 reduced loci of *Cardinium* sp. DF to further explore. First, the NUDIX hydrolase gene LFAMDCML_00833 encoded by the contig gnl|X|LFAMDCML_28 of *Cardinium* sp. TP presented an unexpectedly high percentages of identity, 88.608% and 88.400% with GPDKAJLJ_00014 of Plasmid A and DIOAJDMK_00108 of Plasmid B, respectively, but has no homologue in the genome of *Cardinium* sp. DF (Fig. 2A). Because other genes on the contig gnl|X|LFAMDCML_28 shared high conservation with 2 regions of the genome of *Cardinium* sp. DF (Fig. 2A), this short contig (12,607 bp) was considered part of the chromosomal genome of *Cardinium* sp. TP. Therefore, we proposed that this NUDIX hydrolase gene has decayed in the chromosomal genome of *Cardinium* sp. DF, but interestingly, 2 copies are retained in its plasmids.

Second, the NUBPL iron-transfer P-loop NTPase gene AIMNMLDK_00971 and the hypothetical protein gene AIMNMLDK_00972 of *Cardinium* sp. *Sogatella furcifera* do not have homologues in the genome of *Cardinium* sp. DF, albeit with the conserved synteny in their upstream genes GPMKIAHG_00272-275, but unexpectedly shared high similarities with the 2 genes, DIOAJDMK_00065 and DIOAJDMK_00064 of Plasmid B (Fig. 2B). In the homologous location of the genome of *Cardinium* sp. DF, we identified 3 inverted pairs of highly conserved transposase genes (Fig. 2C), which are possibly related to the molecular mechanism of this reduction, considering that transposable elements can promote prokaryotic genome reduction (15). GPMKIAHG_00272-275 are located on a pair of inverted repeats and GPMKIAHG_00275 was annotated as a transposase (Fig. 2C).

We identified inverted pairs of resolvase genes in the 2 plasmids (Fig. S4). The ones in Plasmid A constitute a transposon in the Tn-3 family, along with the downstream transposase gene GPDKAJLJ_00027 (Fig. S4A), and split a single gene into 2 fragments, GPDKAJLJ_00025 and GPDKAJLJ_00028. Notably, we found in the main chromosome high quality and functional homologous genes to the 5 genes of Plasmid A (GPDKAJLJ_00023-27) (Table S4.1). As for the 2 resolvase genes of Plasmid B (DIOAJDMK_00026 and DIOAJDMK_00027), they do not have homologues in the chromosome (Table S5 and Fig. S4B).

10.1128/msphere.00074-23.16 FIG S4 Dot plots of the inverted repeated regions in the two plasmids of *Cardinium* sp. DF. (A) Dot plot of the region 22,000-27,500 bp of Plasmid A of *Cardinium* sp. DF. (B) Dot plot of the region 22,800-24,200 bp of Plasmid B of *Cardinium* sp. DF. Two pairs of inverted resolvase genes were highlighted in red color. Download FIG S4, TIF file, 0.9 MB. Copyright © 2023 Xiong et al. 2023 Xiong et al. https://creativecommons.org/licenses/by/4.0/ This content is distributed under the terms of the Creative Commons Attribution 4.0 International license.

In intracellular bacteria, including many human pathogens, genome reduction is very common, but its molecular process is not well understood (16–19). As important extrachromosomal elements, plasmids not only participate in the chromosomal genome evolution of intracellular bacteria (20), but also perform reductive evolution like endosymbionts (21). The role of plasmids in genome evolution has been well studied in the bacterial endosymbiont of aphids *Buchnera*, in which some key genes involved in the biosynthesis of tryptophan and leucine were translocated from the chromosome to plasmids, so as to avoid the regulatory control of operons and cause chromosomal genome reduction (22–24). In *Cardinium* endosymbionts, genome reduction was frequently reported (12–14). The plasmids of *Cardinium* sp. Bemisia tabaci cBtQ1 and sp. *Encarsia pergandiella* shared conserved gene syntenies with the genome of *Cardinium* sp. *Sogatella furcifera* that has no plasmid (14), which indicated the gene translocation between chromosome and plasmid. However, more evidence is needed to reveal the role of plasmids in the chromosomal genome evolution.

Our study shows that endogenous plasmids get involved in the ongoing chromosomal genome reduction of *Cardinium* sp. DF. Based on high-coverage long reads, the high quality chromosome and 2 plasmids of *Cardinium* sp. DF were assembled in close circular DNAs, in which their disrupted GC skew features (Fig. 1A and Fig. S3B and D) can be related to the expansion of mobile elements and indicated frequent sequence rearrangement (25). Then, the integrative analyses of the 2 endogenous plasmids in *Cardinium* sp. DF provide insights into the chromosomal genome reduction. The endogenous plasmids play at least 2 roles in the genome evolution of *Cardinium* sp. DF. First, they provide additional copies of genes found in the main chromosome and the structural basis of homologous recombination, which can be used as a substrate for repair mechanisms. Second, when genes decay in the chromosomal genome, their homologous genes remaining in the plasmid can still encode necessary proteins to avoid functional deficits. Collectively, these endogenous plasmids of *Cardinium* sp. DF provide informative snapshots and valuable resources for exploring the ongoing chromosomal genome reduction of *Cardinium* endosymbionts.
