# Supplementary material for: Endogenous Plasmids and Chromosomal Genome Reduction in the Cardinium Endosymbiont of Dermatophagoides farinae
Source: mSphere. 2023 Mar 20;8(2):e00074-23. doi: 10.1128/msphere.00074-23 (PMC10117132; doi:10.1128/msphere.00074-23)
Supplement: TEXT S1 [file msphere.00074-23-s0010.docx]

**Supplemental methods**

**Endogenous Plasmids and Chromosomal Genome Reduction in the *Cardinium* Endosymbiont of *Dermatophagoides farinae***

Qing Xiong^1,2^, Cathy Sin-Hang Fung^1,2^, Xiaojun Xiao^3^, Angel Tsz-Yau Wan^1,2^, Mingqiang Wang^1,2^, Pavel Klimov^4^, Yaning Ren^5^, Kevin Yi Yang^1,2^, Jan Hubert^6^, Yubao Cui^5^, Xiaoyu Liu^3^ *, Stephen Kwok-Wing Tsui^1,2,7^ *

**Affiliations:**

1. School of Biomedical Sciences, The Chinese University of Hong Kong, Hong Kong.
2. Hong Kong Bioinformatics Centre, The Chinese University of Hong Kong, Hong Kong.
3. Shenzhen Key Laboratory of Allergy and Immunology, School of Medicine, Shenzhen University, China.
4. Department of Biological Sciences, Purdue University, West Lafayette, Indiana, USA.
5. Clinical Research Center, The Affiliated Wuxi People’s Hospital of Nanjing Medical University, Wuxi, China.
6. Faculty of Agrobiology, Food and Natural Resources, Czech University of Life Sciences Prague, Prague, Czechia.
7. Centre for Microbial Genomics and Proteomics, The Chinese University of Hong Kong, Hong Kong.

* Correspondence to Professor Stephen Kwok-Wing Tsui, E-mail: [kwtsui@cuhk.edu.hk](mailto:kwtsui@cuhk.edu.hk), Tel: 852-3943 6381, Fax: 852-2603 5123; Professor Xiaoyu Liu, E-mail: [lxy0901@szu.edu.cn](mailto:lxy0901@szu.edu.cn), Tel:86-755-8667 1907, Fax: 86-755-8667 1906.

## *Genome assembly and annotation*

To assemble the genome of *Cardinium* sp. DF, the initial genome assembly was constructed with PacBio long reads using Flye v2.6 (1). Then, further scaffolding was performed by SSPACE Basic v2.0 (2) with paired-end Illumina short reads (3) and SSPACE LongRead v1.1 (4) with PacBio long reads (5) to achieve better continuity. Sequence polishing was finished by Pilon v1.22 (6) with all Illumina short reads. Further scaffolding was performed with SSPACE-LongRead v.1.1 (4) using Oxford Nanopore Technologies (ONT) sequencing reads (7) and gaps were filled with the raw ONT reads using LR_Gapcloser v1.0 (8). The chromosomal genome and two plasmids located in contigs of *Cardinium* sp. DF were *de novo* assembled along with the chromosomal genome of *Dermatophagoides farinae*.

As for the genome of *Cardinium* sp. TP, the assembly was mainly based on next-generation sequencing (NGS) short reads. NGS reads from three studies were collected for assembling draft genomes using SPAdes v3.13.1 (9) in meta mode respectively. From their assembled sequences, three 16S rRNA sequences of *Cardinium* were identified and confirmed as 100% identities. Then combined NGS reads from the three studies were reassembled using SPAdes v3.13.1 (9) in meta mode. The draft genome was further scaffolded using SSPACE Basic v2.0 (2) with paired-end NGS short reads (3) and SSPACE LongRead v1.1 (4) with PacBio long reads (5). Finally, within the assembled sequences, 33 contigs were assigned as from *Cardinium* genus and considered as the genome assembly of *Cardinium* sp. TP.

The genome annotations were performed by Prokka v1.14.6 (10) and more functional annotations were added by eggNOG-mapper (11, 12). The genome and annotation were visualized by the online tool Proksee (<https://proksee.ca/>) (13). To estimate the sequencing coverage, NGS and third-generation sequencing (TGS) reads were mapped to the genome by Bowtie2 v2.3.5.1 (14) and Blasr (15), then transformed, sorted and coverage calculated by Samtools v1.9 (16). Visualization of reads mapping was performed by Integrative Genomics Viewer (IGV) (17). Transposable elements or transposons were annotated by the online tool ISfinder (18).

## *Whole genome alignment*

To understand the sequence similarities, whole genome alignment was performed and visualized using AliTV (<https://alitvteam.github.io/AliTV/d3/AliTV.html>) (19). Additionally, dot plots were generated by Gepard v2.1 (20) to identify and visualize conserved and repeated regions.

## *Comparative genomics*

To explore the evolutionary relationships, comparative genomics analysis was performed among *Cardinium* assemblies (Table S2). All the *Cardinium* genome assemblies were annotated by Prokka v1.14.6 (10) and all the annotated proteomes were assigned into orthogroups (or gene families) based on protein sequence similarities by OrthoFinder v2.5.4 (21). The two plasmids of *Cardinium* sp. DF were not included in this analysis. Then, Venn diagram was performed to identify specific orthogroups using an online tool (<https://bioinformatics.psb.ugent.be/webtools/Venn/>).

Then phylogenetic analysis was performed based on the sequence alignment of 295 single-copy orthogroups. Firstly, protein sequences in the 295 single-copy orthogroups were extracted and aligned by MAFFT (22), then edited in Gblocks (23) with the option ‘-t=p’ to generate sequence alignment of conserved amino-acid residues. Finally, the sequence alignment was used to construct the phylogenetic tree in maximum likelihood algorithm and 100 bootstrap replicates by RAxML v8.2.12 (24) with the options ‘-m PROTCATWAG -f a -# 100’. The other phylogenetic tree based on 16S rRNA (Table S2) was constructed by MEGA v11.0.11 (25) with maximum likelihood (ML) algorithm in the JTT (Jones-Taylor-Thornton) model and 100 bootstrap replicates. The phylogenetic trees were finally edited by the online tool Interactive Tree of Life (iTOL, <https://itol.embl.de/itol.cgi>) (26).

## *Genome reduction analysis*

To explore the protein homology, sequence similarity was analyzed by BLASTP v2.9.0 (27) and sequence alignment was performed by the online tool Clustal Omega (28). Along with the orthogroup assignment, a range of genome reduction loci were identified (Table S5) and further analyzed in gene synteny alignments.

# References

1. Kolmogorov M, Yuan J, Lin Y, Pevzner PA. 2019. Assembly of long, error-prone reads using repeat graphs. Nature Biotechnology 37:540-546.

2. Boetzer M, Henkel CV, Jansen HJ, Butler D, Pirovano W. 2010. Scaffolding pre-assembled contigs using SSPACE. Bioinformatics 27:578-579.

3. Bentley DR, Balasubramanian S, Swerdlow HP, Smith GP, Milton J, Brown CG, Hall KP, Evers DJ, Barnes CL, Bignell HR, Boutell JM, Bryant J, Carter RJ, Keira Cheetham R, Cox AJ, Ellis DJ, Flatbush MR, Gormley NA, Humphray SJ, Irving LJ, Karbelashvili MS, Kirk SM, Li H, Liu X, Maisinger KS, Murray LJ, Obradovic B, Ost T, Parkinson ML, Pratt MR, Rasolonjatovo IM, Reed MT, Rigatti R, Rodighiero C, Ross MT, Sabot A, Sankar SV, Scally A, Schroth GP, Smith ME, Smith VP, Spiridou A, Torrance PE, Tzonev SS, Vermaas EH, Walter K, Wu X, Zhang L, Alam MD, Anastasi C, et al. 2008. Accurate whole human genome sequencing using reversible terminator chemistry. Nature 456:53-9.

4. Boetzer M, Pirovano W. 2014. SSPACE-LongRead: scaffolding bacterial draft genomes using long read sequence information. BMC Bioinformatics 15:211.

5. Eid J, Fehr A, Gray J, Luong K, Lyle J, Otto G, Peluso P, Rank D, Baybayan P, Bettman B. 2009. Real-time DNA sequencing from single polymerase molecules. Science 323:133-138.

6. Walker BJ, Abeel T, Shea T, Priest M, Abouelliel A, Sakthikumar S, Cuomo CA, Zeng Q, Wortman J, Young SK. 2014. Pilon: an integrated tool for comprehensive microbial variant detection and genome assembly improvement. PloS one 9:e112963.

7. Deamer D, Akeson M, Branton D. 2016. Three decades of nanopore sequencing. Nature Biotechnology 34:518-524.

8. Xu G-C, Xu T-J, Zhu R, Zhang Y, Li S-Q, Wang H-W, Li J-T. 2018. LR_Gapcloser: a tiling path-based gap closer that uses long reads to complete genome assembly. GigaScience 8.

9. Bankevich A, Nurk S, Antipov D, Gurevich AA, Dvorkin M, Kulikov AS, Lesin VM, Nikolenko SI, Pham S, Prjibelski AD. 2012. SPAdes: a new genome assembly algorithm and its applications to single-cell sequencing. Journal of computational biology 19:455-477.

10. Seemann T. 2014. Prokka: rapid prokaryotic genome annotation. Bioinformatics 30:2068-2069.

11. Cantalapiedra CP, Hernández-Plaza A, Letunic I, Bork P, Huerta-Cepas J. 2021. eggNOG-mapper v2: Functional Annotation, Orthology Assignments, and Domain Prediction at the Metagenomic Scale. Molecular Biology and Evolution 38:5825-5829.

12. Huerta-Cepas J, Szklarczyk D, Heller D, Hernández-Plaza A, Forslund SK, Cook H, Mende DR, Letunic I, Rattei T, Jensen LJ, von Mering C, Bork P. 2019. eggNOG 5.0: a hierarchical, functionally and phylogenetically annotated orthology resource based on 5090 organisms and 2502 viruses. Nucleic Acids Res 47:D309-d314.

13. Grant JR, Stothard P. 2008. The CGView Server: a comparative genomics tool for circular genomes. Nucleic acids research 36:W181-W184.

14. Langmead B, Salzberg SL. 2012. Fast gapped-read alignment with Bowtie 2. Nat Methods 9:357-9.

15. Chaisson MJ, Tesler G. 2012. Mapping single molecule sequencing reads using basic local alignment with successive refinement (BLASR): application and theory. BMC Bioinformatics 13:238.

16. Li H, Handsaker B, Wysoker A, Fennell T, Ruan J, Homer N, Marth G, Abecasis G, Durbin R, Subgroup GPDP. 2009. The Sequence Alignment/Map format and SAMtools. Bioinformatics 25:2078-2079.

17. Robinson JT, Thorvaldsdóttir H, Winckler W, Guttman M, Lander ES, Getz G, Mesirov JP. 2011. Integrative genomics viewer. Nat Biotechnol 29:24-6.

18. Siguier P, Perochon J, Lestrade L, Mahillon J, Chandler M. 2006. ISfinder: the reference centre for bacterial insertion sequences. Nucleic Acids Res 34:D32-6.

19. Ankenbrand MJ, Hohlfeld S, Hackl T, Förster F. 2017. AliTV—interactive visualization of whole genome comparisons. PeerJ Computer Science 3:e116.

20. Krumsiek J, Arnold R, Rattei T. 2007. Gepard: a rapid and sensitive tool for creating dotplots on genome scale. Bioinformatics 23:1026-1028.

21. Emms D, Kelly S. 2018. OrthoFinder2: fast and accurate phylogenomic orthology analysis from gene sequences. BioRxiv 466201.

22. Katoh K, Misawa K, Kuma Ki, Miyata T. 2002. MAFFT: a novel method for rapid multiple sequence alignment based on fast Fourier transform. Nucleic Acids Research 30:3059-3066.

23. Castresana J. 2000. Selection of Conserved Blocks from Multiple Alignments for Their Use in Phylogenetic Analysis. Molecular Biology and Evolution 17:540-552.

24. Stamatakis A. 2014. RAxML version 8: a tool for phylogenetic analysis and post-analysis of large phylogenies. Bioinformatics (Oxford, England) 30:1312-1313.

25. Tamura K, Stecher G, Kumar S. 2021. MEGA11: Molecular Evolutionary Genetics Analysis Version 11. Molecular Biology and Evolution 38:3022-3027.

26. Letunic I, Bork P. 2021. Interactive Tree Of Life (iTOL) v5: an online tool for phylogenetic tree display and annotation. Nucleic Acids Research 49:W293-W296.

27. McGinnis S, Madden TL. 2004. BLAST: at the core of a powerful and diverse set of sequence analysis tools. Nucleic Acids Research 32:W20-W25.

28. Sievers F, Wilm A, Dineen D, Gibson TJ, Karplus K, Li W, Lopez R, McWilliam H, Remmert M, Söding J. 2011. Fast, scalable generation of high‐quality protein multiple sequence alignments using Clustal Omega. Molecular systems biology 7:539.
