# Supplementary material for: Endogenous Plasmids and Chromosomal Genome Reduction in the Cardinium Endosymbiont of Dermatophagoides farinae
Source: mSphere. 2023 Mar 20;8(2):e00074-23. doi: 10.1128/msphere.00074-23 (PMC10117132; doi:10.1128/msphere.00074-23)
Supplement: TABLE S1 [file msphere.00074-23-s0005.docx]

|  | *Cardinium* sp. DF ^a^ | *Cardinium* sp. DF UM *^c^* | *Cardinium* sp. TP ^a^ |
| --- | --- | --- | --- |
| Genome size | **1,259,597 bp** | **1,258,868 bp** | **914,750 bp** |
| Genome assembly |  |  |  |
| Assembly level | 1 contig | 1 contig | 33 contigs |
| Assembled DNA type | Circular DNA | Linear DNA | Linear DNA |
| Assembly completeness ^b^ | 76.4% | 66.9% | 73.6% |
| Genome annotation |  |  |  |
| Protein-coding genes | 1,198 | 1,349 | 841 |
| rRNA genes | 3 | 3 | 3 |
| tRNA genes | 35 | 35 | 33 |
| tmRNA gene | 1 | 1 | 1 |
| Annotation completeness ^b^ | 77.7% | 68.9% | 76.4% |

a Two *de novo* assembled genomes. GenBank accessions: CP101107.1 for the chromosome of *Cardinium* sp. DF and JANAVR000000000.1 for the genome of *Cardinium* sp. TP.

b The completeness was assessed by BUSCO v3.1.0 with the database bacteria_odb9.

c The longest contig NZ_VMBH01000001.1 that was considered as the main chromosome. GenBank accession: GCF_007559345.1.
