# Supplementary figures and images for: Endogenous Plasmids and Chromosomal Genome Reduction in the Cardinium Endosymbiont of Dermatophagoides farinae
Source: mSphere. 2023 Mar 20;8(2):e00074-23. doi: 10.1128/msphere.00074-23 (PMC10117132; doi:10.1128/msphere.00074-23)

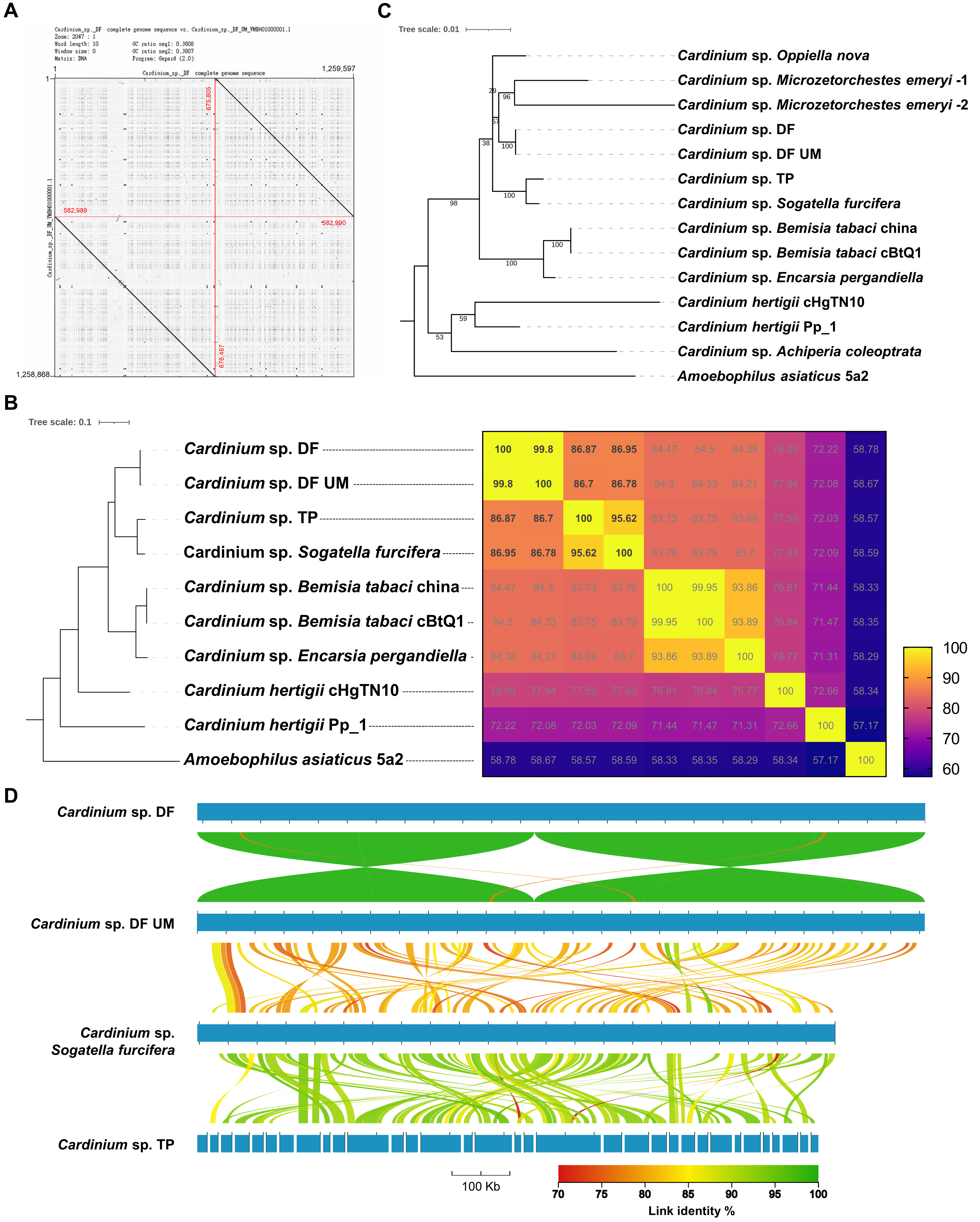

Supplement: FIG S1 [file msphere.00074-23-s0001.tif]

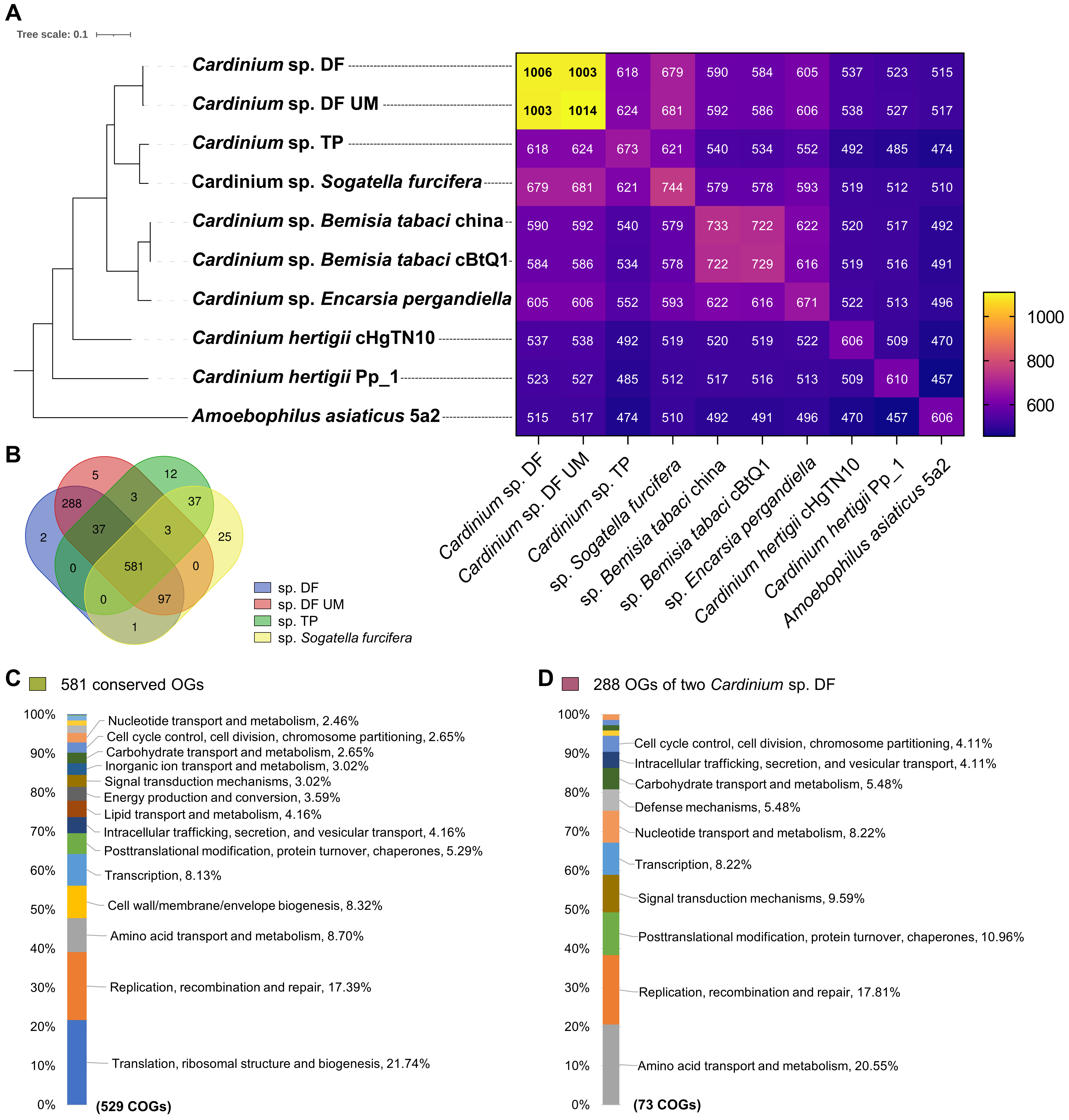

Supplement: FIG S2 [file msphere.00074-23-s0002.tif]

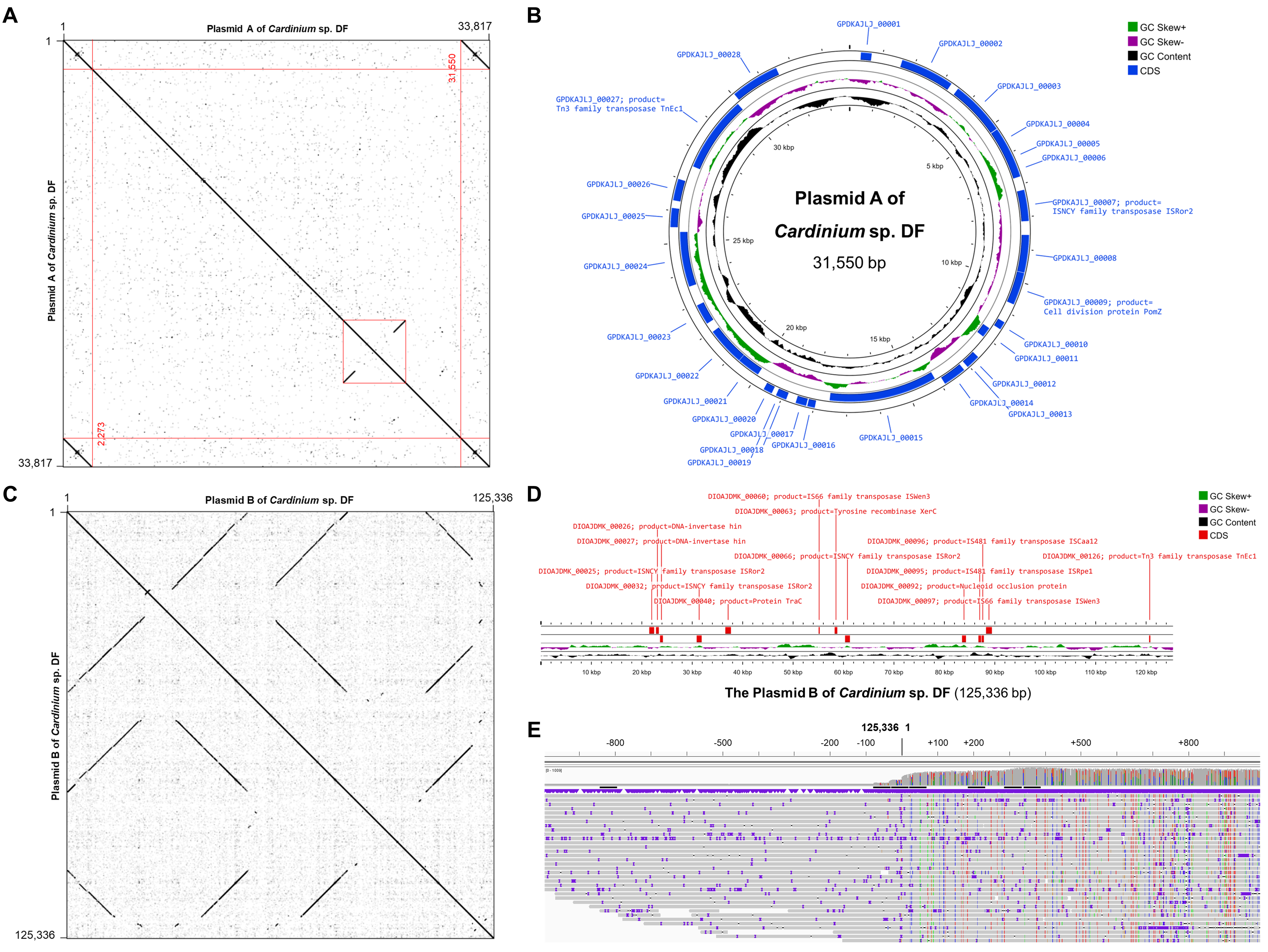

Supplement: FIG S3 [file msphere.00074-23-s0003.tif]

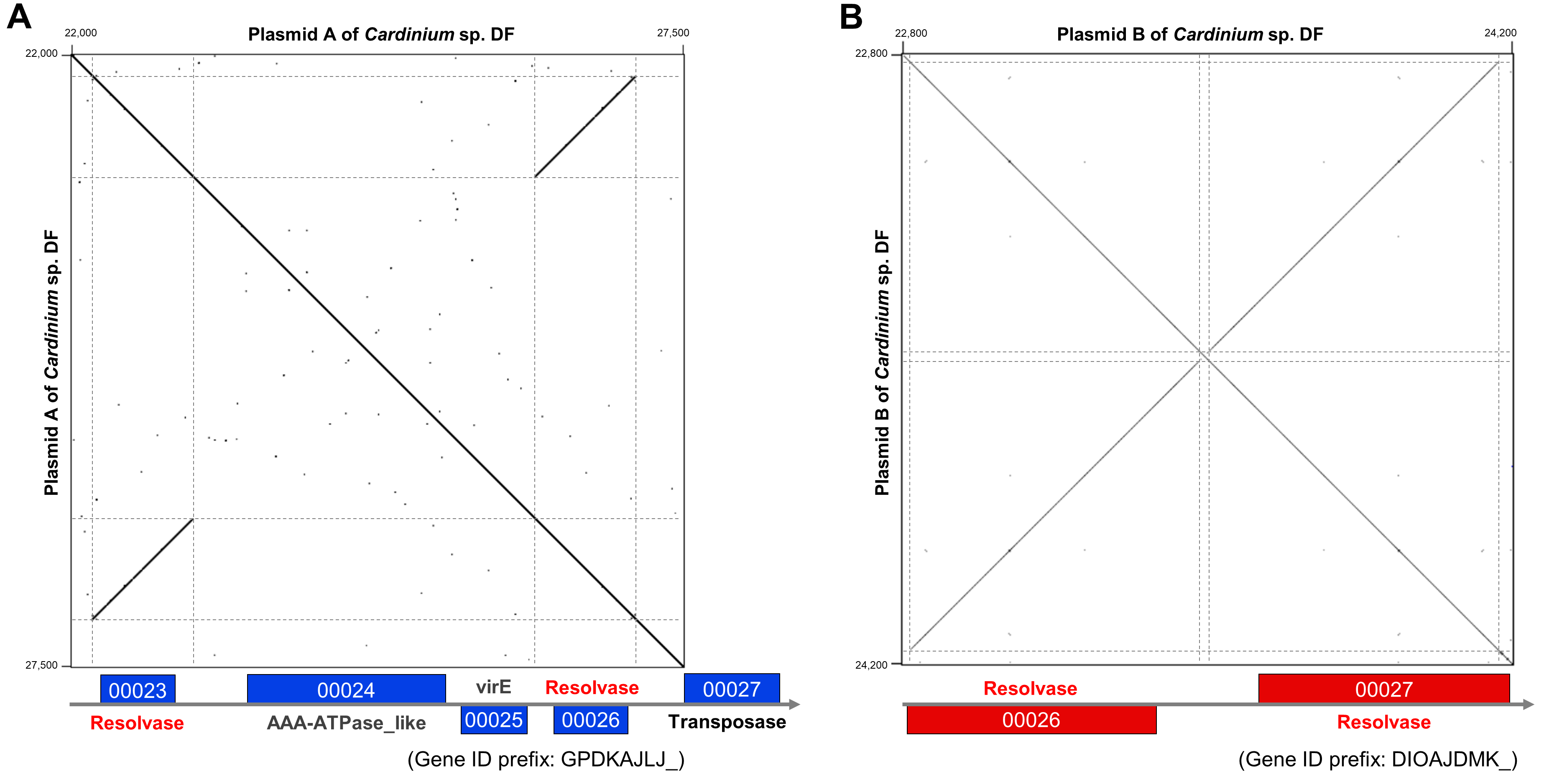

Supplement: FIG S4 [file msphere.00074-23-s0004.tif]
