# Supplementary material for: Endogenous Plasmids and Chromosomal Genome Reduction in the Cardinium Endosymbiont of Dermatophagoides farinae
Source: mSphere. 2023 Mar 20;8(2):e00074-23. doi: 10.1128/msphere.00074-23 (PMC10117132; doi:10.1128/msphere.00074-23)
Supplement: TABLE S2 [file msphere.00074-23-s0006.docx]

| **Name used in this study** | **Host species** | **GenBank accession** | **Genome size (bp)** | **Number of contig/scaffold** |
| --- | --- | --- | --- | --- |
| *Cardinium* sp. DF | *Dermatophagoides farinae* | CP101107.1 ^a^ | 1,259,597 | 1 |
| *Cardinium* sp. DF UM | *Dermatophagoides farinae* | GCA_007559345.1 | 1,258,868 | 1 |
| *Cardinium* sp. TP | *Tyrophagus putrescentiae* | JANAVR000000000.1 ^a^ | 914,750 | 33 |
| Cardinium sp. *Sogatella furcifera* | *Sogatella furcifera* | GCA_003351905.1 | 1,103,593 | 1 |
| *Cardinium* sp. *Bemisia tabaci* china | *Bemisia tabaci* | GCA_004300865.1 | 1,012,588 | 3 |
| *Cardinium* sp. *Bemisia tabaci* cBtQ1 | *Bemisia tabaci* | GCA_000689375.1 | 996,809 | 11 |
| *Cardinium* sp. *Encarsia pergandiella* | *Encarsia pergandiella* | GCA_000304455.1 | 944,930 | 2 |
| *Cardinium hertigii* cHgTN10 | *Heterodera glycines* | GCA_003176915.1 | 1,193,042 | 1 |
| *Cardinium hertigii* Pp_1 | *Pratylenchus penetrans* | GCA_003788695.1 | 1,358,212 | 27 |
| *Amoebophilus asiaticus* 5a2 | *Acanthamoeba* sp. TUMSJ-321 | GCA_000020565.1 | 1,884,364 | 1 |
| *Cardinium* sp. *Oppiella nova* | *Oppiella nova* | AY279414 ^b^ |  |  |
| *Cardinium* sp. *Achiperia coleoptrata* | *Achiperia coleoptrata* | MG889457 ^b^ |  |  |
| *Cardinium* sp. *Microzetorchestes emeryi* -1 | *Microzetorchestes emeryi* | MG889458 ^b^ |  |  |
| *Cardinium* sp. *Microzetorchestes emeryi* -2 | *Microzetorchestes emeryi* | MG889459 ^b^ |  |  |

a *De novo* assembled genomes in this study.

b Partial 16S rRNA sequences.
