# Supplementary material for: Endogenous Plasmids and Chromosomal Genome Reduction in the Cardinium Endosymbiont of Dermatophagoides farinae
Source: mSphere. 2023 Mar 20;8(2):e00074-23. doi: 10.1128/msphere.00074-23 (PMC10117132; doi:10.1128/msphere.00074-23)
Supplement: TABLE S3 [file msphere.00074-23-s0007.docx]

**Table S3.1. EggNOG-mapper output of protein sequences of Plasmid A**

| **query** | **Description** | **PFAMs** | **Preferred name** |
| --- | --- | --- | --- |
| ^*^GPDKAJLJ_00002 | Psort location Cytoplasmic, score 8.96 | AAA-ATPase_like,PDDEXK_9 | - |
| ^*^GPDKAJLJ_00003 | TraM recognition site of TraD and TraG | T4SS-DNA_transf,TraG-D_C,TrwB_AAD_bind | - |
| GPDKAJLJ_00004 | Relaxase/Mobilisation nuclease domain | Relaxase | bmgA |
| GPDKAJLJ_00006 | - | - | - |
| ^#^GPDKAJLJ_00007 | putative transposase, YhgA-like | Transposase_31 | - |
| GPDKAJLJ_00008 | - | - | - |
| GPDKAJLJ_00009 | AAA domain | AAA_31 | - |
| GPDKAJLJ_00010 | toxin SymE, type I toxin-antitoxin system | SymE_toxin | - |
| GPDKAJLJ_00011 | helix-turn-helix XRE-family like proteins | HTH_3 | - |
| ^*^GPDKAJLJ_00012 | GDP-mannose mannosyl hydrolase activity | NUDIX | - |
| GPDKAJLJ_00013 | GDP-mannose mannosyl hydrolase activity | NUDIX | - |
| GPDKAJLJ_00014 | NUDIX hydrolase | NUDIX | - |
| ^*^GPDKAJLJ_00015 | symporter activity | HD_4,HTH_18,SSF | - |
| ^#^GPDKAJLJ_00023 | Resolvase | Resolvase | - |
| ^*^GPDKAJLJ_00024 | - | AAA-ATPase_like,HTH_3,PDDEXK_9 | - |
| ^*^GPDKAJLJ_00025 | P-loop ATPase and inactivated | VirE | virE |
| ^#^GPDKAJLJ_00026 | Resolvase | Resolvase | - |
| ^#^GPDKAJLJ_00027 | Tn3 transposase DDE domain | DDE_Tnp_Tn3,DUF4158 | - |
| ^*^GPDKAJLJ_00028 | P-loop ATPase and inactivated | VirE | virE |

* These genes have high-similarity homologs (bit score 150 as cutoff) in the chromosome of *Cardinium* sp. DF.

# These genes are DNA rearrangement-related genes and have high-similarity homologs (bit score 150 as cutoff) in the chromosome of *Cardinium* sp. DF.

**Table S3.2. EggNOG-mapper output of protein sequences of Plasmid B**

| query | Description | | PFAMs | Preferred name |
| --- | --- | --- | --- | --- |
| DIOAJDMK_00001 | DNA primase activity | | DUF3991,DnaB_C,Toprim_2,Toprim_3,zf-CHC2 | - |
| ^*^DIOAJDMK_00002 | DNA primase activity | | DUF3991,DnaB_C,Toprim_2,Toprim_3,zf-CHC2 | - |
| DIOAJDMK_00004 | DNA primase activity | | PriCT_2,Prim-Pol,Toprim_2,Toprim_3,zf-CHC2 | - |
| ^*^DIOAJDMK_00006 | plasmid recombination enzyme | | Mob_Pre | - |
| ^*^DIOAJDMK_00009 | TraM recognition site of TraD and TraG | | T4SS-DNA_transf,TraG-D_C,TrwB_AAD_bind | - |
| ^*^DIOAJDMK_00010 | response to abiotic stimulus | | Ank_2,Ank_3,Ank_4,Ank_5,Rhodanese | - |
| ^*^DIOAJDMK_00016 | type IV secretory pathway VirB4 | | DUF3875,DUF87 | - |
| ^*^DIOAJDMK_00017 | - |  | - | - |
| ^*^DIOAJDMK_00018 | conjugation | | VirB8 | trbF |
| ^*^DIOAJDMK_00021 | conjugal transfer protein TraM | | Transposon_TraM | - |
| ^#^DIOAJDMK_00025 | putative transposase, YhgA-like | | Transposase_31 | - |
| DIOAJDMK_00026 | Resolvase, N terminal domain | | HTH_7,Resolvase | - |
| DIOAJDMK_00027 | Resolvase, N terminal domain | | HTH_7,Resolvase | - |
| DIOAJDMK_00028 | N-acetylmuramoyl-L-alanine amidase | | Amidase_2,PG_binding_1 | amiD |
| DIOAJDMK_00029 | NLPC_P60 stabilising domain, N term | | NLPC_P60,N_NLPC_P60,SH3_6,SH3_7 | - |
| ^*^DIOAJDMK_00030 | symporter activity | | HD_4,HTH_18,SSF | - |
| ^*^DIOAJDMK_00031 | symporter activity | | HD_4,HTH_18,SSF | - |
| ^#^DIOAJDMK_00032 | putative transposase, YhgA-like | | Transposase_31 | - |
| ^*^DIOAJDMK_00035 | TIGRFAM Bacteroides conjugative transposon TraM protein | | Transposon_TraM | - |
| ^*^DIOAJDMK_00037 | conjugation | | VirB8 | trbF |
| ^*^DIOAJDMK_00038 | - |  | - | - |
| ^*^DIOAJDMK_00040 | type IV secretory pathway VirB4 | | DUF3875,DUF87 | - |
| ^*^DIOAJDMK_00041 | type IV secretory pathway VirB4 | | DUF3875,DUF87 | - |
| ^*^DIOAJDMK_00048 | response to abiotic stimulus | | Ank_2,Ank_3,Ank_4,Ank_5,Rhodanese | - |
| ^*^DIOAJDMK_00049 | response to abiotic stimulus | | Ank_2,Ank_3,Ank_4,Ank_5,Rhodanese | - |
| ^*^DIOAJDMK_00051 | TraM recognition site of TraD and TraG | | T4SS-DNA_transf,TraG-D_C,TrwB_AAD_bind | - |
| ^*^DIOAJDMK_00052 | TraM recognition site of TraD and TraG | | T4SS-DNA_transf,TraG-D_C,TrwB_AAD_bind | - |
| ^*^DIOAJDMK_00054 | plasmid recombination enzyme | | Mob_Pre | - |
| ^*^DIOAJDMK_00056 | P-loop ATPase and inactivated | | VirE | virE |
| DIOAJDMK_00057 | P-loop ATPase and inactivated | | VirE | virE |
| DIOAJDMK_00060 | Transposase IS66 family | | DDE_Tnp_IS66,zf-IS66 | - |
| DIOAJDMK_00061 | to TIGR01784 | | PDDEXK_2 | - |
| DIOAJDMK_00062 | N-terminal domain of reverse transcriptase | | GIIM,RVT_1,RVT_N | - |
| ^*^DIOAJDMK_00063 | belongs to the 'phage' integrase family | | Phage_int_SAM_5,Phage_integrase | - |
| DIOAJDMK_00065 | NUBPL iron-transfer P-loop NTPase | | AAA_31,CbiA | - |
| ^#^DIOAJDMK_00066 | putative transposase, YhgA-like | | Transposase_31 | - |
| ^*^DIOAJDMK_00069 | conjugal transfer protein TraM | | Transposon_TraM | - |
| ^*^DIOAJDMK_00071 | conjugation | | VirB8 | trbF |
| ^*^DIOAJDMK_00072 | - |  | - | - |
| ^*^DIOAJDMK_00073 | type IV secretory pathway VirB4 | | DUF3875,DUF87 | - |
| ^*^DIOAJDMK_00078 | response to abiotic stimulus | | Ank_2,Ank_3,Ank_4,Ank_5,Rhodanese | - |
| ^*^DIOAJDMK_00079 | TraM recognition site of TraD and TraG | | T4SS-DNA_transf,TraG-D_C,TrwB_AAD_bind | - |
| DIOAJDMK_00082 | plasmid recombination enzyme | | Mob_Pre | - |
| DIOAJDMK_00087 | DNA primase activity | | PriCT_2,Prim-Pol,Toprim_2,Toprim_3,zf-CHC2 | - |
| ^*^DIOAJDMK_00088 | DNA primase activity | | DUF3991,DnaB_C,Toprim_2,Toprim_3,zf-CHC2 | - |
| ^*^DIOAJDMK_00089 | P-loop ATPase and inactivated | | VirE | virE |
| ^*^DIOAJDMK_00090 | P-loop ATPase and inactivated | | VirE | virE |
| DIOAJDMK_00091 | cellulose biosynthesis protein BcsQ | | AAA_31 | - |
| DIOAJDMK_00092 | ParB-like nuclease domain | | ParBc | - |
| ^*^DIOAJDMK_00093 | GDP-mannose mannosyl hydrolase activity | | NUDIX | - |
| DIOAJDMK_00094 | domain of unknown function (DUF4277) | | DDE_Tnp_1,DUF4277 | - |
| ^*^DIOAJDMK_00095 | winged helix-turn helix | | HTH_29,rve,rve_3 | - |
| ^*^DIOAJDMK_00096 | winged helix-turn helix | | HTH_29,rve,rve_3 | - |
| DIOAJDMK_00097 | Transposase IS66 family | | DDE_Tnp_IS66,zf-IS66 | - |
| DIOAJDMK_00098 | - |  | - | - |
| DIOAJDMK_00103 | response to abiotic stimulus | | Ank,Ank_2,Ank_3,Ank_4,Ank_5 | - |
| ^*^DIOAJDMK_00106 | symporter activity | | HD_4,HTH_18,SSF | - |
| ^*^DIOAJDMK_00107 | symporter activity | | HD_4,HTH_18,SSF | - |
| DIOAJDMK_00108 | NUDIX hydrolase | | NUDIX | - |
| ^*^DIOAJDMK_00109 | GDP-mannose mannosyl hydrolase activity | | NUDIX | - |
| DIOAJDMK_00111 | ATPase MipZ | | AAA_31,MipZ | - |
| ^*^DIOAJDMK_00113 | P-loop ATPase and inactivated | | VirE | virE |
| ^*^DIOAJDMK_00114 | DNA primase activity | | PriCT_2,Prim-Pol,Toprim_2,Toprim_3,zf-CHC2 | - |
| ^*^DIOAJDMK_00116 | plasmid recombination enzyme | | Mob_Pre | - |
| ^*^DIOAJDMK_00118 | TraM recognition site of TraD and TraG | | T4SS-DNA_transf,TraG-D_C,TrwB_AAD_bind | - |
| ^*^DIOAJDMK_00119 | response to abiotic stimulus | | Ank_2,Ank_3,Ank_4,Ank_5,Rhodanese | - |
| ^#^DIOAJDMK_00124 | Resolvase |  | Resolvase | - |
| ^*^DIOAJDMK_00125 | domain of unknown function (DUF4158) | | DDE_Tnp_Tn3,DUF4158 | - |
| ^#^DIOAJDMK_00126 | Tn3 transposase DDE domain | | DDE_Tnp_Tn3,DUF4158 | - |
| DIOAJDMK_00128 | PD-(D/E)XK nuclease family transposase | | PDDEXK_2 | - |
| DIOAJDMK_00129 | plasmid maintenance | | AAA_31,MipZ | - |
| DIOAJDMK_00130 | Resolvase |  | Resolvase | - |

* These genes have high-similarity homologs (bit score 150 as cutoff) in the chromosome of *Cardinium* sp. DF.

# These genes are DNA rearrangement-related genes and have high-similarity homologs (bit score 150 as cutoff) in the chromosome of *Cardinium* sp. DF.
