# Supplementary material for: Endogenous Plasmids and Chromosomal Genome Reduction in the Cardinium Endosymbiont of Dermatophagoides farinae
Source: mSphere. 2023 Mar 20;8(2):e00074-23. doi: 10.1128/msphere.00074-23 (PMC10117132; doi:10.1128/msphere.00074-23)
Supplement: TABLE S4 [file msphere.00074-23-s0008.docx]

**Table S4.1. BLASTP output of annotated protein sequences of Plasmid A**

| **query id** | **subject id** | **% identity** | **alignment length** | **mismatches** | **gap opens** | **q. start** | **q. end** | **s. start** | **s. end** | **evalue** | **bit score** |
| --- | --- | --- | --- | --- | --- | --- | --- | --- | --- | --- | --- |
| GPDKAJLJ_00027 | GPMKIAHG_00026 | 100 | 818 | 0 | 0 | 1 | 818 | 88 | 905 | 0 | 1691 |
| GPDKAJLJ_00015 | GPMKIAHG_00420 | 70.766 | 992 | 284 | 2 | 3 | 993 | 4 | 990 | 0 | 1415 |
| GPDKAJLJ_00024 | GPMKIAHG_00626 | 77.304 | 586 | 133 | 0 | 1 | 586 | 1 | 586 | 0 | 942 |
| GPDKAJLJ_00002 | GPMKIAHG_00240 | 76.867 | 549 | 116 | 2 | 1 | 538 | 1 | 549 | 0 | 871 |
| GPDKAJLJ_00028 | GPMKIAHG_00251 | 62.702 | 496 | 181 | 3 | 1 | 494 | 1 | 494 | 0 | 629 |
| GPDKAJLJ_00007 | GPMKIAHG_00518 | 82.555 | 321 | 44 | 2 | 7 | 315 | 3 | 323 | 0 | 542 |
| GPDKAJLJ_00026 | GPMKIAHG_00891 | 100 | 219 | 0 | 0 | 1 | 219 | 1 | 219 | 1.76E-167 | 455 |
| GPDKAJLJ_00023 | GPMKIAHG_00834 | 99.543 | 219 | 1 | 0 | 1 | 219 | 1 | 219 | 3.56E-166 | 452 |
| GPDKAJLJ_00022 | GPMKIAHG_00003 | 57.25 | 400 | 158 | 6 | 22 | 411 | 1 | 397 | 2.39E-146 | 417 |
| GPDKAJLJ_00025 | GPMKIAHG_00293 | 64.583 | 192 | 67 | 1 | 1 | 191 | 38 | 229 | 3.54E-83 | 241 |
| GPDKAJLJ_00003 | GPMKIAHG_01234 | 29.893 | 562 | 341 | 15 | 20 | 551 | 81 | 619 | 1.01E-65 | 227 |
| GPDKAJLJ_00021 | GPMKIAHG_00253 | 45.802 | 262 | 116 | 7 | 4 | 242 | 13 | 271 | 5.49E-69 | 209 |
| GPDKAJLJ_00012 | GPMKIAHG_00418 | 83.673 | 98 | 16 | 0 | 1 | 98 | 47 | 144 | 6.06E-58 | 170 |
| GPDKAJLJ_00019 | GPMKIAHG_00295 | 86.364 | 44 | 6 | 0 | 1 | 44 | 145 | 188 | 4.04E-23 | 82.4 |
| GPDKAJLJ_00013 | GPMKIAHG_00418 | 81.818 | 44 | 8 | 0 | 1 | 44 | 1 | 44 | 4.97E-24 | 82.4 |
| GPDKAJLJ_00020 | GPMKIAHG_00295 | 48.837 | 86 | 39 | 1 | 3 | 88 | 14 | 94 | 5.08E-20 | 76.3 |
| GPDKAJLJ_00009 | GPMKIAHG_00596 | 27.602 | 221 | 146 | 6 | 58 | 272 | 3 | 215 | 1.44E-17 | 76.3 |
| GPDKAJLJ_00022 | GPMKIAHG_00003 | 32.609 | 184 | 103 | 7 | 67 | 233 | 220 | 399 | 6.87E-16 | 73.9 |
| GPDKAJLJ_00001 | GPMKIAHG_01231 | 37.5 | 88 | 55 | 0 | 12 | 99 | 14 | 101 | 3.04E-18 | 69.3 |
| GPDKAJLJ_00018 | GPMKIAHG_00295 | 60.938 | 64 | 25 | 0 | 12 | 75 | 187 | 250 | 7.26E-16 | 64.7 |
| GPDKAJLJ_00016 | GPMKIAHG_00162 | 39.655 | 58 | 34 | 1 | 14 | 71 | 210 | 266 | 9.38E-07 | 39.7 |

**Table S4.2. BLASTP output of GPDKAJLJ_00027**

| **query id** | **subject id** | **% identity** | **alignment length** | **mismatches** | **gap opens** | **q. start** | **q. end** | **s. start** | **s. end** | **evalue** | **bit score** |
| --- | --- | --- | --- | --- | --- | --- | --- | --- | --- | --- | --- |
| GPDKAJLJ_00027 | GPMKIAHG_00026 | 100 | 818 | 0 | 0 | 1 | 818 | 88 | 905 | 0 | 1691 |
| GPDKAJLJ_00027 | GPMKIAHG_00833 | 99.51 | 818 | 4 | 0 | 1 | 818 | 88 | 905 | 0 | 1680 |
| GPDKAJLJ_00027 | GPMKIAHG_00658 | 97.99 | 797 | 16 | 0 | 22 | 818 | 1 | 797 | 0 | 1610 |
| GPDKAJLJ_00027 | GPMKIAHG_00290 | 99.72 | 707 | 2 | 0 | 112 | 818 | 1 | 707 | 0 | 1458 |
| GPDKAJLJ_00027 | GPMKIAHG_01011 | 99.55 | 664 | 3 | 0 | 155 | 818 | 1 | 664 | 0 | 1372 |
| GPDKAJLJ_00027 | GPMKIAHG_00299 | 98.85 | 435 | 5 | 0 | 384 | 818 | 1 | 435 | 0 | 894 |
| GPDKAJLJ_00027 | GPMKIAHG_00627 | 98.55 | 414 | 6 | 0 | 405 | 818 | 1 | 414 | 0 | 851 |
| GPDKAJLJ_00027 | GPMKIAHG_00893 | 99.75 | 394 | 1 | 0 | 1 | 394 | 1 | 394 | 0 | 809 |
| GPDKAJLJ_00027 | GPMKIAHG_00628 | 98.24 | 397 | 7 | 0 | 1 | 397 | 88 | 484 | 0 | 803 |
| GPDKAJLJ_00027 | GPMKIAHG_00298 | 97.87 | 376 | 8 | 0 | 1 | 376 | 88 | 463 | 0 | 759 |
| GPDKAJLJ_00027 | GPMKIAHG_00894 | 98.72 | 235 | 3 | 0 | 584 | 818 | 45 | 279 | 2.00E-165 | 481 |
| GPDKAJLJ_00027 | GPMKIAHG_00894 | 59.78 | 92 | 31 | 2 | 393 | 484 | 2 | 87 | 4.00E-22 | 94 |
| GPDKAJLJ_00027 | GPMKIAHG_01113 | 98.14 | 161 | 3 | 0 | 1 | 161 | 150 | 310 | 7.00E-104 | 322 |
| GPDKAJLJ_00027 | GPMKIAHG_01110 | 98.14 | 161 | 3 | 0 | 1 | 161 | 150 | 310 | 1.00E-103 | 322 |
| GPDKAJLJ_00027 | GPMKIAHG_01012 | 94.74 | 133 | 7 | 0 | 20 | 152 | 3 | 135 | 5.00E-79 | 249 |
| GPDKAJLJ_00027 | GPMKIAHG_00076 | 94.57 | 129 | 5 | 1 | 1 | 127 | 88 | 216 | 2.00E-76 | 246 |
| GPDKAJLJ_00027 | GPMKIAHG_00291 | 100 | 109 | 0 | 0 | 1 | 109 | 88 | 196 | 5.00E-69 | 224 |
| GPDKAJLJ_00027 | GPMKIAHG_00010 | 36.15 | 296 | 174 | 7 | 424 | 710 | 1 | 290 | 2.00E-49 | 173 |
| GPDKAJLJ_00027 | GPMKIAHG_00011 | 25.41 | 362 | 258 | 6 | 9 | 360 | 15 | 374 | 3.00E-29 | 116 |
| GPDKAJLJ_00027 | GPMKIAHG_00009 | 37.14 | 105 | 66 | 0 | 711 | 815 | 12 | 116 | 1.00E-17 | 76.6 |
| GPDKAJLJ_00027 | GPMKIAHG_01013 | 92.59 | 27 | 2 | 0 | 1 | 27 | 88 | 114 | 2.00E-10 | 55.8 |

**Table S4.3. BLASTP output of annotated protein sequences of Plasmid B**

| **query id** | **subject id** | **% identity** | **alignment length** | **mismatches** | **gap opens** | **q. start** | **q. end** | **s. start** | **s. end** | **evalue** | **bit score** |
| --- | --- | --- | --- | --- | --- | --- | --- | --- | --- | --- | --- |
| DIOAJDMK_00073 | GPMKIAHG_00283 | 90.858 | 711 | 65 | 0 | 1 | 711 | 1 | 711 | 0 | 1343 |
| DIOAJDMK_00016 | GPMKIAHG_00283 | 90.577 | 711 | 67 | 0 | 1 | 711 | 1 | 711 | 0 | 1335 |
| DIOAJDMK_00116 | GPMKIAHG_01232 | 89.342 | 638 | 68 | 0 | 1 | 638 | 1 | 638 | 0 | 1184 |
| DIOAJDMK_00054 | GPMKIAHG_01232 | 89.342 | 638 | 68 | 0 | 1 | 638 | 1 | 638 | 0 | 1184 |
| DIOAJDMK_00030 | GPMKIAHG_00375 | 57.352 | 1027 | 429 | 6 | 10 | 1032 | 10 | 1031 | 0 | 1161 |
| DIOAJDMK_00118 | GPMKIAHG_01234 | 92.845 | 601 | 43 | 0 | 17 | 617 | 17 | 617 | 0 | 1154 |
| DIOAJDMK_00079 | GPMKIAHG_01234 | 92.845 | 601 | 43 | 0 | 17 | 617 | 17 | 617 | 0 | 1154 |
| DIOAJDMK_00006 | GPMKIAHG_01232 | 86.614 | 635 | 85 | 0 | 1 | 635 | 1 | 635 | 0 | 1151 |
| DIOAJDMK_00009 | GPMKIAHG_01234 | 92.487 | 599 | 45 | 0 | 1 | 599 | 19 | 617 | 0 | 1146 |
| DIOAJDMK_00010 | GPMKIAHG_00267 | 89.949 | 587 | 59 | 0 | 1 | 587 | 1 | 587 | 0 | 1090 |
| DIOAJDMK_00119 | GPMKIAHG_01234 | 84.588 | 558 | 80 | 2 | 60 | 614 | 597 | 1151 | 0 | 966 |
| DIOAJDMK_00078 | GPMKIAHG_01234 | 84.588 | 558 | 80 | 2 | 60 | 614 | 597 | 1151 | 0 | 966 |
| DIOAJDMK_00056 | GPMKIAHG_00251 | 84.404 | 545 | 85 | 0 | 47 | 591 | 1 | 545 | 0 | 956 |
| DIOAJDMK_00106 | GPMKIAHG_00420 | 75.702 | 605 | 146 | 1 | 3 | 606 | 386 | 990 | 0 | 933 |
| DIOAJDMK_00113 | GPMKIAHG_00021 | 85.857 | 502 | 71 | 0 | 84 | 585 | 1 | 502 | 0 | 870 |
| DIOAJDMK_00041 | GPMKIAHG_00283 | 88.032 | 376 | 45 | 0 | 1 | 376 | 1 | 376 | 0 | 696 |
| DIOAJDMK_00049 | GPMKIAHG_00267 | 83.815 | 346 | 56 | 0 | 1 | 346 | 130 | 475 | 0 | 608 |
| DIOAJDMK_00034 | GPMKIAHG_00273 | 96.382 | 304 | 11 | 0 | 1 | 304 | 1 | 304 | 0 | 584 |
| DIOAJDMK_00022 | GPMKIAHG_00273 | 94.737 | 304 | 16 | 0 | 1 | 304 | 1 | 304 | 0 | 577 |
| DIOAJDMK_00068 | GPMKIAHG_00273 | 92.763 | 304 | 22 | 0 | 1 | 304 | 1 | 304 | 0 | 567 |
| DIOAJDMK_00051 | GPMKIAHG_01234 | 92.491 | 293 | 22 | 0 | 1 | 293 | 323 | 615 | 0 | 558 |
| DIOAJDMK_00076 | GPMKIAHG_01236 | 95.941 | 271 | 11 | 0 | 5 | 275 | 1 | 271 | 0 | 553 |
| DIOAJDMK_00121 | GPMKIAHG_01236 | 95.941 | 271 | 11 | 0 | 5 | 275 | 1 | 271 | 0 | 549 |
| DIOAJDMK_00032 | GPMKIAHG_00275 | 80.665 | 331 | 52 | 1 | 1 | 331 | 1 | 319 | 0 | 546 |
| DIOAJDMK_00066 | GPMKIAHG_00518 | 80.122 | 327 | 53 | 2 | 5 | 323 | 1 | 323 | 0 | 533 |
| DIOAJDMK_00025 | GPMKIAHG_00401 | 79.817 | 327 | 54 | 2 | 1 | 327 | 1 | 315 | 0 | 531 |
| DIOAJDMK_00021 | GPMKIAHG_00272 | 92.708 | 288 | 21 | 0 | 1 | 288 | 1 | 288 | 0 | 516 |
| DIOAJDMK_00040 | GPMKIAHG_00283 | 91.176 | 272 | 24 | 0 | 1 | 272 | 447 | 718 | 1.8E-180 | 514 |
| DIOAJDMK_00035 | GPMKIAHG_00272 | 88.542 | 288 | 31 | 1 | 1 | 286 | 1 | 288 | 5.3E-179 | 490 |
| DIOAJDMK_00069 | GPMKIAHG_00272 | 87.241 | 290 | 35 | 1 | 1 | 290 | 1 | 288 | 1.4E-178 | 489 |
| DIOAJDMK_00017 | GPMKIAHG_00285 | 93.727 | 271 | 17 | 0 | 1 | 271 | 1 | 271 | 1.3E-169 | 465 |
| DIOAJDMK_00072 | GPMKIAHG_00285 | 92.251 | 271 | 21 | 0 | 1 | 271 | 1 | 271 | 2.4E-165 | 454 |
| DIOAJDMK_00124 | GPMKIAHG_00834 | 98.174 | 219 | 4 | 0 | 1 | 219 | 1 | 219 | 2.3E-165 | 450 |
| DIOAJDMK_00088 | GPMKIAHG_00251 | 70.508 | 295 | 87 | 0 | 1 | 295 | 52 | 346 | 3.2E-151 | 430 |
| DIOAJDMK_00107 | GPMKIAHG_00420 | 61.429 | 350 | 129 | 2 | 1 | 349 | 40 | 384 | 5.6E-141 | 420 |
| DIOAJDMK_00012 | GPMKIAHG_01236 | 86.486 | 222 | 30 | 0 | 5 | 226 | 1 | 222 | 1.7E-145 | 402 |
| DIOAJDMK_00125 | GPMKIAHG_01113 | 97.98 | 198 | 4 | 0 | 1 | 198 | 1 | 198 | 3.1E-143 | 398 |
| DIOAJDMK_00018 | GPMKIAHG_00690 | 81.735 | 219 | 40 | 0 | 1 | 219 | 1 | 219 | 1E-137 | 380 |
| DIOAJDMK_00013 | GPMKIAHG_01237 | 82.028 | 217 | 39 | 0 | 46 | 262 | 7 | 223 | 5.3E-131 | 380 |
| DIOAJDMK_00037 | GPMKIAHG_00690 | 85.096 | 208 | 31 | 0 | 13 | 220 | 12 | 219 | 5.2E-137 | 379 |
| DIOAJDMK_00071 | GPMKIAHG_00690 | 85.096 | 208 | 31 | 0 | 13 | 220 | 12 | 219 | 1.8E-136 | 377 |
| DIOAJDMK_00044 | GPMKIAHG_01237 | 83.105 | 219 | 37 | 0 | 181 | 399 | 7 | 225 | 1.9E-131 | 376 |
| DIOAJDMK_00052 | GPMKIAHG_00265 | 84.762 | 210 | 32 | 0 | 8 | 217 | 93 | 302 | 2.9E-129 | 370 |
| DIOAJDMK_00077 | GPMKIAHG_01235 | 94.22 | 173 | 10 | 0 | 1 | 173 | 1 | 173 | 3.9E-123 | 339 |
| DIOAJDMK_00120 | GPMKIAHG_01235 | 94.22 | 173 | 10 | 0 | 1 | 173 | 1 | 173 | 1.2E-122 | 338 |
| DIOAJDMK_00011 | GPMKIAHG_01235 | 91.329 | 173 | 15 | 0 | 1 | 173 | 1 | 173 | 9.7E-121 | 333 |
| DIOAJDMK_00089 | GPMKIAHG_00021 | 82.162 | 185 | 33 | 0 | 1 | 185 | 238 | 422 | 4.5E-111 | 321 |
| DIOAJDMK_00095 | GPMKIAHG_00002 | 95.57 | 158 | 7 | 0 | 1 | 158 | 1 | 158 | 1.6E-108 | 309 |
| DIOAJDMK_00036 | GPMKIAHG_00689 | 81.013 | 158 | 30 | 0 | 1 | 158 | 1 | 158 | 1.05E-94 | 271 |
| DIOAJDMK_00096 | GPMKIAHG_00002 | 94.615 | 130 | 7 | 0 | 1 | 130 | 211 | 340 | 1.58E-93 | 270 |
| DIOAJDMK_00070 | GPMKIAHG_00689 | 81.013 | 158 | 30 | 0 | 1 | 158 | 1 | 158 | 4.64E-94 | 270 |
| DIOAJDMK_00038 | GPMKIAHG_00285 | 97.656 | 128 | 3 | 0 | 1 | 128 | 144 | 271 | 3.56E-90 | 258 |
| DIOAJDMK_00031 | GPMKIAHG_01172 | 78.846 | 156 | 33 | 0 | 5 | 160 | 220 | 375 | 4.79E-88 | 258 |
| DIOAJDMK_00109 | GPMKIAHG_00418 | 81.25 | 144 | 27 | 0 | 1 | 144 | 1 | 144 | 1.26E-88 | 250 |
| DIOAJDMK_00043 | GPMKIAHG_00280 | 74.85 | 167 | 42 | 0 | 1 | 167 | 144 | 310 | 5.59E-85 | 249 |
| DIOAJDMK_00093 | GPMKIAHG_00418 | 82.734 | 139 | 24 | 0 | 10 | 148 | 6 | 144 | 4.19E-86 | 244 |
| DIOAJDMK_00063 | GPMKIAHG_00257 | 87.302 | 126 | 16 | 0 | 1 | 126 | 1 | 126 | 1.75E-82 | 237 |
| DIOAJDMK_00019 | GPMKIAHG_00689 | 82.171 | 129 | 23 | 0 | 1 | 129 | 31 | 159 | 9.73E-79 | 225 |
| DIOAJDMK_00127 | GPMKIAHG_00274 | 92.982 | 114 | 8 | 0 | 1 | 114 | 1 | 114 | 5.05E-76 | 217 |
| DIOAJDMK_00114 | GPMKIAHG_00023 | 96.078 | 102 | 4 | 0 | 1 | 102 | 1 | 102 | 6.68E-72 | 209 |
| DIOAJDMK_00048 | GPMKIAHG_01234 | 79.365 | 126 | 26 | 0 | 1 | 126 | 1026 | 1151 | 4.18E-65 | 208 |
| DIOAJDMK_00020 | GPMKIAHG_00271 | 94.495 | 109 | 6 | 0 | 1 | 109 | 1 | 109 | 9.66E-73 | 207 |
| DIOAJDMK_00015 | GPMKIAHG_00282 | 89.72 | 107 | 11 | 0 | 1 | 107 | 1 | 107 | 3.84E-72 | 205 |
| DIOAJDMK_00067 | GPMKIAHG_00261 | 87.069 | 116 | 15 | 0 | 1 | 116 | 1 | 116 | 3.70E-70 | 201 |
| DIOAJDMK_00074 | GPMKIAHG_00282 | 87.85 | 107 | 13 | 0 | 1 | 107 | 1 | 107 | 3.74E-69 | 197 |
| DIOAJDMK_00002 | GPMKIAHG_00251 | 73.077 | 130 | 35 | 0 | 1 | 130 | 52 | 181 | 4.19E-62 | 194 |
| DIOAJDMK_00090 | GPMKIAHG_00252 | 75.735 | 136 | 31 | 1 | 1 | 134 | 35 | 170 | 2.16E-64 | 189 |
| DIOAJDMK_00047 | GPMKIAHG_01235 | 87.129 | 101 | 13 | 0 | 1 | 101 | 1 | 101 | 9.68E-63 | 184 |
| DIOAJDMK_00039 | GPMKIAHG_00285 | 92.623 | 122 | 9 | 0 | 1 | 122 | 1 | 122 | 2.16E-59 | 179 |
| DIOAJDMK_00058 | GPMKIAHG_00020 | 47.847 | 209 | 104 | 1 | 1 | 204 | 1 | 209 | 2.59E-56 | 173 |
| DIOAJDMK_00045 | GPMKIAHG_01236 | 95.181 | 83 | 4 | 0 | 5 | 87 | 1 | 83 | 1.39E-55 | 168 |
| DIOAJDMK_00059 | GPMKIAHG_00019 | 41.852 | 270 | 153 | 3 | 1 | 268 | 1 | 268 | 2.42E-52 | 167 |
| DIOAJDMK_00126 | GPMKIAHG_01110 | 98.795 | 83 | 1 | 0 | 1 | 83 | 230 | 312 | 2.72E-54 | 166 |
| DIOAJDMK_00081 | GPMKIAHG_01232 | 93.902 | 82 | 5 | 0 | 1 | 82 | 1 | 82 | 2.07E-50 | 163 |
| DIOAJDMK_00042 | GPMKIAHG_00281 | 96.203 | 79 | 3 | 0 | 1 | 79 | 1 | 79 | 3.51E-53 | 155 |
