# Supplementary material for: Endogenous Plasmids and Chromosomal Genome Reduction in the Cardinium Endosymbiont of Dermatophagoides farinae
Source: mSphere. 2023 Mar 20;8(2):e00074-23. doi: 10.1128/msphere.00074-23 (PMC10117132; doi:10.1128/msphere.00074-23)
Supplement: TABLE S5 [file msphere.00074-23-s0009.docx]

| **Gene ID** | **Location** | **Description** | **Best-match gene** | **% identity** | **E-value** |
| --- | --- | --- | --- | --- | --- |
| GPDKAJLJ_00014 | Plasmid A | NUDIX hydrolase | LFAMDCML_00833  *Cardinium* sp. TP | 88.608% | 2.37E-158 |
| DIOAJDMK_00026 | Plasmid B | Resolvase, N terminal domain | AIMNMLDK_00049  *Cardinium* sp. *Sogatella furcifera* | 90.957% | 5.31E-117 |
| DIOAJDMK_00027 | Plasmid B | Resolvase, N terminal domain | AIMNMLDK_00049  *Cardinium* sp. *Sogatella furcifera* | 91.667% | 8.10E-121 |
| DIOAJDMK_00062 | Plasmid B | N-terminal domain of reverse transcriptase | AIMNMLDK_00186  *Cardinium* sp. *Sogatella furcifera* | 38.889% | 7.86E-71 |
| DIOAJDMK_00064 | Plasmid B | Hypothetical protein | AIMNMLDK_00972  *Cardinium* sp. *Sogatella furcifera* | 95.181% | 1.25E-56 |
| DIOAJDMK_00065 | Plasmid B | NUBPL iron-transfer P-loop NTPase | AIMNMLDK_00971  *Cardinium* sp. *Sogatella furcifera* | 96.685% | 6.95E-130 |
| DIOAJDMK_00108 | Plasmid B | NUDIX hydrolase | LFAMDCML_00833  *Cardinium* sp. TP | 88.400% | 3.46E-168 |
| DIOAJDMK_00131 | Plasmid B | Hypothetical protein | AIMNMLDK_00978  *Cardinium* sp. *Sogatella furcifera* | 56.574% | 4.06E-94 |
| DIOAJDMK_00134 | Plasmid B | Hypothetical protein | AIMNMLDK_00979  *Cardinium* sp. *Sogatella furcifera* | 61.905% | 3.11E-34 |
